# Self-assembly of DNA nanostructure containing cell-specific aptamer as a precise drug delivery system for cancer therapy in non-small cell lung cancer

**DOI:** 10.1186/s12951-022-01701-5

**Published:** 2022-11-19

**Authors:** Ning Wang, Chang Yu, Tingting Xu, Dan Yao, Lingye Zhu, Zhifa Shen, Xiaoying Huang

**Affiliations:** 1 Division of Pulmonary Medicine, the First Affiliated Hospital of Wenzhou Medical University, Key Laboratory of Heart and Lung, Wenzhou, Zhejiang 325000 China; 2 Intervention Department, the First Affiliated Hospital of Wenzhou Medical University, Wenzhou, Zhejiang 325000 China; 3 Key Laboratory of Laboratory Medicine, Ministry of Education of China, and Zhejiang Provincial Key Laboratory of Medical Genetics, School of Laboratory Medicine and Life Sciences, Wenzhou Medical University, Wenzhou, Zhejiang 325035 China

**Keywords:** DNA nanotechnology, Non-small cell lung cancer, Self-assembly, Precise drug delivery, Antineoplastic efficacy

## Abstract

**Background:**

As the most common subtype in lung cancer, the precise and efficient treatment for non-small cell lung cancer (NSCLC) remains an outstanding challenge owing to early metastasis and poor prognosis. Chemotherapy, the most commonly used treatment modality, is a difficult choice for many cancer patients due to insufficient drug accumulation in tumor sites and severe systemic side-effects. In this study, we constructed a cell-specific aptamer-modified DNA nanostructure (Apt-NS) as a targeting drug delivery system achieving the precision therapy for lung cancer.

**Methods:**

The synthesis of DNA nanostructure and its stability were evaluated using gel electrophoresis. The targeting properties and internalization mechanism were investigated via flow cytometry and confocal analyses. Drug loading, release, and targeted drug delivery were determined by fluorescence detection, Zeta potentials assay, and confocal imaging. CCK8 assays, colony formation, cell apoptosis, metastasis analyses and in vivo experiments were conducted to assess the biological functions of DNA nanostructure.

**Results:**

Self-assembled DNA nanoparticles (Apt-NS) had excellent stability to serum and DNase I and the ability to specifically recognize A549 cells. Upon specific binding, the drug-loaded nanoparticles (Apt-NS-DOX) were internalized into target cells by clathrin-mediated endocytosis. Subsequently, DOX could be released from Apt-NS-DOX based on the degradation of the lysosome. Apt-NS-DOX exerted significant suppression of cell proliferation, invasion and migration, and also enhanced cell apoptosis due to the excellent performance of drug delivery and intracellular release, while maintaining a superior biosafety. In addition, the antitumor effects of Apt-NS-DOX were further confirmed using in vivo models.

**Conclusions:**

Our study provided cell-specific aptamer-modified DNA nanostructures as a drug-delivery system targeting A549 cells, which could precisely and efficiently transport chemotherapeutic drug into tumor cells, exerting enhanced antineoplastic efficacy. These findings highlight that DNA nanostructure serving as an ideal drug delivery system in cancer treatment appears great promise in biomedical applications.

**Supplementary Information:**

The online version contains supplementary material available at 10.1186/s12951-022-01701-5.

## Introduction

According to the latest report of Global Cancer Statistics 2020, lung cancer has become the second most common diagnosed cancer (11.4% of the whole cases) and a leading contributor to cancer-related deaths (18.0% of the whole cancer deaths) in humans worldwide, with a low 5-year survival rate (~ 20%) [1, 2]. Non-small cell lung cancer (NSCLC) is the primary histologic type accounting for 85% of total lung cancer cases [3]. Currently, the incidence and mortality rates of lung cancer are still highest in China. In recent years, the development of driver genes mutations investigation and targeted therapies have reformed the therapeutic approaches to lung cancer patients [4, 5]. Meanwhile, immunotherapy has grown rapidly and emerged as a significant treatment option for many lung cancer patients. Despite the application of targeted therapy and immunotherapy, chemotherapy, especially combined chemotherapy, remains the most commonly used and effective treatment strategy for lung cancer.

Doxorubicin (DOX), one of the anthracyclines, has been considered as a routine clinical application in cancer chemotherapy for a wide range of malignancies, including lung cancer [6, 7]. The antitumor mechanisms of DOX on the one hand selectively intercalate into DNA and inhibit the activity of topoisomerase II resulting in the DNA damage response (DDR) [8]. On the other hand, DOX can disrupt proteins-mediated DNA damage repair by forming a stable DOX-topoisomerase II complex [9]. Recently, many patients presented poor overall response rates to DOX accompanied by cardiotoxic side effect resulting in heart failure [10, 11]. Generally, chemotherapy is a difficult choice for many patients with cancer owing to undesired and severe side-effects [12, 13]. Therefore, it is urgent to develop an efficient therapeutic strategy for improving the sensitivity of chemotherapy for cancer patients, while reducing systemic adverse reactions.

Recent years have seen great development in the drug delivery carriers based on nanotechnology in the application of novel chemotherapy strategies, of which nucleic acid-based functional nanomaterials (NAFN), including DNA origami, DNA nanoflower, DNA tetrahedrons and so on, can realize gene or drug loading through the DNA self-assembly. DNA nanostructures serving as effective drug delivery vehicles have several potential advantages, such as low immunogenicity, inherent biocompatibility, favorable structural stability, and flexible programmability [14], which has hold great promise in the field of cancer research, involving in diagnosis, drug or gene delivery, and targeted therapy [15–18]. DNA aptamers can be screened from a large library of oligonucleotides using SELEX (Systematic Evolution of Ligands by Exponential enrichment) method, and has been applied in molecular diagnostics and therapeutics due to high affinity and specificity characteristics [19]. Based on cell-SELEX, DNA aptamers can distinguish target cells from nontarget cells, realizing effectively specific recognition of certain cancer cells. The aptamer has been widely applied in tumor targeted chemotherapy owing to their efficient and specific tumor recognition ability [20–22]. In previous studies, DNA aptamer S6 selected by cell-SELEX could effectively recognize A549 cells [23], and had been applied in the construction of targeted drug delivery system [24–27]. As a solution, DNA nanostructure emerged combining the characteristics of prolonged blood circulation time, enhanced tumor targeting and excellent biosafety, thus could protect chemotherapy drugs from early blood attenuation. Therefore, DNA nanostructures as drug delivery system require to be accurately designed and optimized for efficient drug loading, enhancing the capacity for antitumor treatment.

**Scheme 1:**
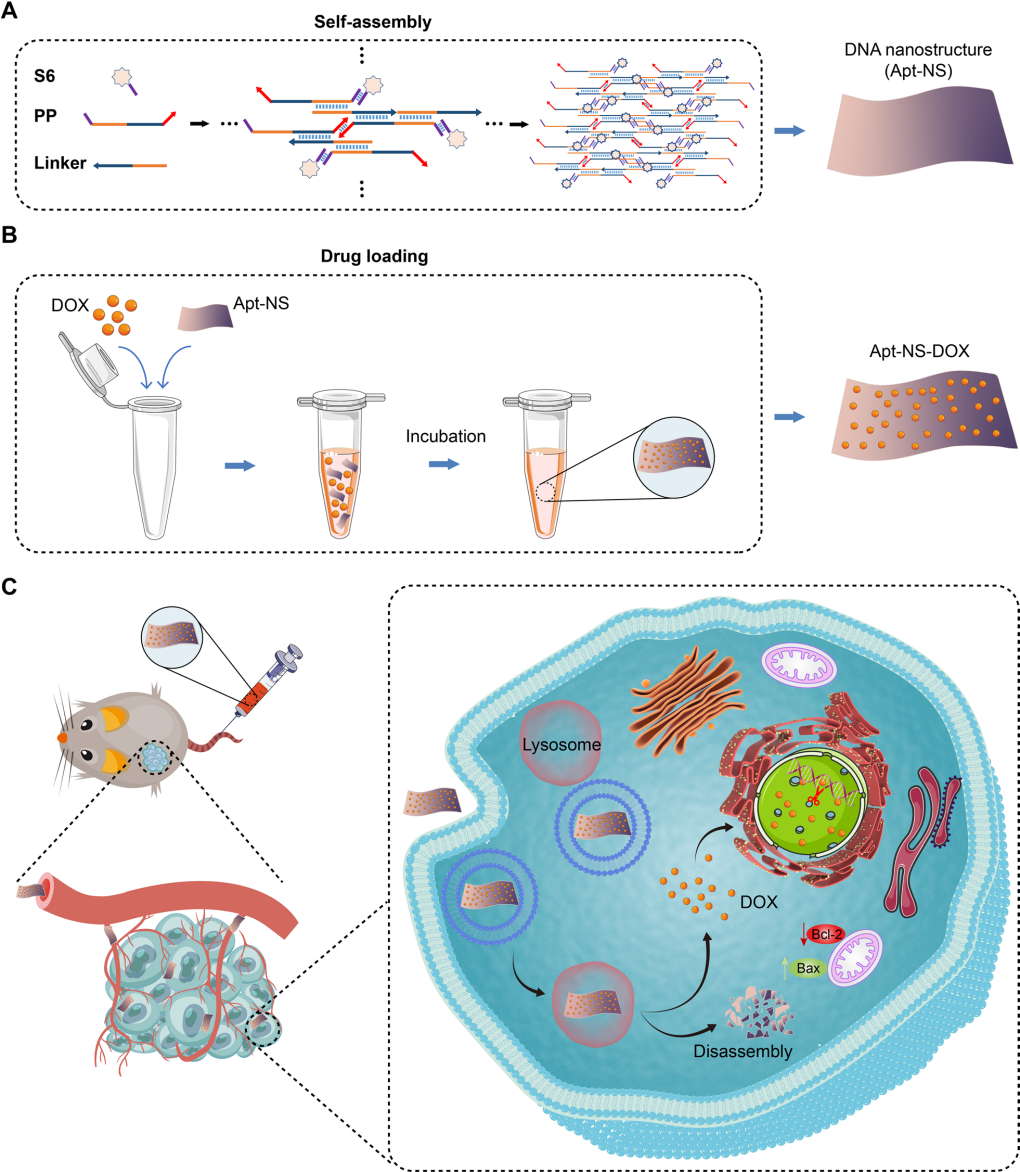
Schematic illustration of the synthesis of Apt-NS, Apt-NS-DOX, and the antitumor effects. **A** Preparation scheme of Apt-NS using three single-strand DNA, including S6 (aptamer), PP (palindrome probe), and Linker. **B** Drug loading process of Apt-NS for the formation of Apt-NS-DOX. **C** After intravenous injection, Apt-NS-DOX was specifically internalized into tumor cells, and then released its cargo (DOX) by lysosomal catabolism. The DOX could enter into the nucleus and induce DNA damage leading to cell apoptosis

Herein, to realize the precise delivery and release of the chemotherapeutic drug (DOX), we constructed a suitable size and high stability DNA nanostructure (Apt-NS) containing cell-specific aptamer based on the assembly of three simple single-strand DNA. The experimental results on cellular uptake indicated that Apt-NS exerted stronger affinity to target cells (A549 cells) than nontarget cells (HELF cells). Through efficiently loading DOX, Apt-NS-DOX significantly improved the ability of precise delivery of chemotherapy drugs, and exhibited significant tumor suppression effects (Scheme 1). Our research revealed that DNA nanomaterials could be applied as powerful therapeutic platform for cancer treatment. Taken together, this study constructed a simple effective DNA nanocarrier as precise drug delivery system to realize greatly enhanced antineoplastic efficacy and decreased side effects.

## Materials and methods

### Materials

All oligonucleotides designed in our research were provided by Sangon Biotech Co., Ltd. (Shanghai, China), and the detailed sequences information can be observed in Additional file 1: Table S1. All single-strand DNA (ssDNA) were dissolved in TE buffer and stored at 4 °C for subsequent experiments. The 10 × TM buffer (pH = 8.0) were prepared using Tris–HCl (10 mM) and MgCl_2_·6H_2_O (50 mM). Doxorubicin was purchased from Aladdin (Shanghai, China). Tris–HCl, MgCl_2_·6H_2_O, and SYBR Green I nucleic acid dyestuff was obtained from Solarbio Science & Technology Co., Ltd (Beijing, China). TE buffer, DAPI solution, DNase I enzyme, and LysoTracker Green were obtained from Beyotime Biotechnology (Shanghai, China).

### Assembly of Apt-NS

Three ssDNA, including S6 (aptamer), PP (palindrome probe), and Linker, were mixed into TM buffer with equal molar ratio (final concentration of 800 nM each), heated at 90 °C for 5 min, and then the reaction slowly cooled down to room temperature. The synthesis of DNA nanostructure was accomplished after annealing process.

### Gel electrophoresis analysis

Polyacrylamide gel electrophoresis (PAGE) was conducted to validate the assembly of DNA nanostructure. The mixture of DNA samples (10 μL) and working solution (SYBR Green I and 6 × loading buffer) (2 μL) was analyzed via 8% PAGE at 80 V for 60 min with 0.5 × TBE buffer. Since the bands were stained with SYBR Green I, the resulting images could be observed via the gel imaging system (Bio-Rad, USA).

### Morphological characterization of Apt-NS

Transmission electron microscopy (TEM, Japan) was utilized to determine the size of DNA nanostructure. For Atomic Force Microscopy (AFM) observation, the Apt-NS samples were diluted tenfold and 10–20 μL were pipetted onto freshly cleaved mica flakes. After adsorption for 15 min, the size and morphology of Apt-NS was measured using AFM (Bruker, Germany), followed by data analysis using the Nanoscope analysis 1.7 software.

### Stability analysis

To test the serum stability, different samples such as Linker, PP, and Apt-NS were incubated with 10% FBS at 37 °C with setting different time points (0, 2, 6, 12, and 24 h). After incubation, final products were analyzed via 8% PAGE. To test the DNase I stability, Apt-NS samples were incubated with DNase I (2 U/mL) at 37 °C with setting different time points (0, 2, 6, 12, and 24 h). After incubation, EDTA (final concentration of 2.5 mM) was applied to terminate the enzymatic reaction with immediately heating at 65 °C for 10 min. Then, the final products were determined using 8% PAGE as described above. Quantitative assessment of the stability was conducted based on gel electrophoresis images via Image J software.

### Cell culture

Human NSCLC cells (A549) and human embryonic lung fibroblast cells (HELF) were acquired from Institute of Biochemistry and Cell Biology, Chinese Academy of Science (Shanghai, China), and cultivated separately in F-12 k (Zhong Qiao Xin Zhou Biotechnology Co.,Ltd, Shanghai, China) and high glucose Dulbecco’s Modified Eagle’s media (DMEM) (Gibco, USA) culture medium mixed with 10% fetal bovine serum (FBS), and 1% penicillin/streptomycin at 37 °C in the incubator (Thermo, USA) containing 5% CO_2_.

### Cellular uptake assays by flow cytometry and confocal laser scanning microscopy (CLSM)

For flow cytometry analysis, A549 and HELF cells were inoculated into six-well plates and cultivated for 24 h before the experiment. The A549 and HELF cells were incubated with Cy5-labeled Apt-NS (40 μL) in serum free medium (500 μL) for 4 h at 37 °C in 5% CO_2_. After incubation, the collected cells following trypsin digestion were washed, centrifugated, and then resuspended in 500 μL PBS for detection via flow cytometry (Beckman, USA). The results were processed by FlowJo V10 software. For CLSM experiments, the slides of interest cells were prepared before 24 h. The cells were then incubated with Cy5-labeled Apt-NS using the same treatment as flow cytometry. Subsequently, the cells were subjected to washing with PBS, fixation with 4% paraformaldehyde (15 min), and staining with DAPI (1 μg/mL, 5 min), followed by observing and photographing using CLSM (Nikon, Japan).

### Endocytosis pathways of Apt-NS

To investigate the mechanisms of Apt-NS internalization, A549 cells were separately incubated with three endocytosis inhibitors [28] including sucrose (154 mg/mL), amiloride (133 μg/mL), and nystatin (15 μg/mL) for 1 h. Then, the cells were continued to be incubated with medium mixed with both Cy5-labeled Apt-NS and endocytosis inhibitors for 2 h. Finally, the cells were detected using flow cytometry and CLSM imaging after the treatment with standard procedure.

### Intracellular colocalization analysis of Apt-NS

The A549 cells were incubated with Cy5-labeled Apt-NS following a standard procedure same to cellular uptake analysis. At the two time points (2 and 4 h), the cells were incubated with LysoTracker Green (1 μM) for 45 min at 37 °C for lysosomes specific staining. Subsequently, the cells were imaged using CLSM after the treatment with standard procedure. Image J software was applied to perform the colocalization analysis.

### Drug loading and release

In order to realize the drug loading of DNA nanomaterials, DOX (2 µM final concentration) was separately added into different concentrations of Apt-NS (the final concentration as follows: 0, 0.625, 1.25, 1.875, 2.5, 3.125, 3.75, 4.375 and 5.0 nM), and then the resulting solution was diluted to 200 µL with 1 × TM buffer and incubated at room temperature overnight. As the DNA nanostructures (Apt-NS) were loaded with DOX, Apt-NS-DOX were obtained. We first carried out the fluorescence spectra measurements via F-7000 fluorescence spectrophotometer (Hitachi, Japan) (λex = 488 nm, λem = 520–700 nm). Furthermore, zeta potentials of Apt-NS and Apt-NS-DOX were measured using particle size analyzer (Malvern, United Kingdom). For drug release, Apt-NS-DOX was treated with or without DNase I enzyme (20 U/mL), respectively, and F-7000 instrument was utilized to detect the fluorescence signals of DOX released from Apt-NS-DOX.

### Targeting delivery of Apt-NS-DOX

To assess the capacity of intracellular drug delivery, the slides of interest cells were separately incubated with fresh serum-free medium containing free DOX and Cy5-labeled Apt-NS-DOX (the same drug concentration 4 μM) at 37 °C for different time points (0.5, 1, 2 and 4 h). Subsequently, the cells were imaged using CLSM after the treatment with standard procedure.

### Cell viability assay

The cytotoxicity of Apt-NS, DOX, and Apt-NS-DOX was evaluated by Cell Counting Kit-8 (CCK-8) assay. Specifically, A549 and HELF cells (1 × 10^4^ cells/well) were planted into 96-well plates and cultured at 37 °C for 24 h before the experiment. For Apt-NS, the cells were incubated with fresh serum-free medium (100 μL) containing different concentrations (0 nM, 0.547 nM, 1.094 nM, 2.188 nM, 4.375 nM, and 8.75 nM) of Apt-NS for 24 h. For free drug, the cells were incubated with fresh serum-free medium (100 μL) containing different concentrations (0 μM, 0.125 μM, 0.25 μM, 0.5 μM, 1 μM, 2 μM, and 4 μM) of DOX for 24 h. For Apt-NS-DOX, the cells were incubated with fresh serum-free medium (100 μL) containing different equivalent concentrations (0 μM, 0.125 μM, 0.25 μM, 0.5 μM, 1 μM, 2 μM, and 4 μM) of DOX loaded into Apt-NS for 24 h. And then the cells each well were incubated with the mixture containing 10 μL CCK-8 (APExBIO, USA) and 100 μL fresh serum-free medium for 3 h at 37 °C. Finally, the cell viability was assessed by measuring the absorbance at 450 nm via a multifunctional microplate reader (Thermo, USA).

### Colony formation assay

The A549 cells were treated with Apt-NS, DOX (2 μM), Apt-NS-DOX (equivalent DOX concentration) for 24 h, with setting the blank control group. Subsequently, the correspondingly treated cells (1000 cells per well) were planted into six-well plates and cultivated at 37 °C in 5% CO_2_ for one week. Fresh medium was changed every two days. Finally, the colonies were subjected to fixation with 4% paraformaldehyde (10 min) and staining with crystal violet (20 min, Beyotime, China), and then counted for statistical analysis.

### Western blot analysis

A549 cells were planted into 60 mm petri dishes and cultivated for 24 h, and then incubated with fresh serum-free medium containing Apt-NS, DOX (2 μM), Apt-NS-DOX (equivalent DOX concentration) for 24 h, with setting the blank control group. After incubation, culture medium was removed, and the cells were further cultured for 24 h in fresh medium. Then, the total proteins were extracted according to standard protocols. After quantification by BCA protein assay kit (Beyotime, China) and denaturation by heating at 100 °C for 10 min. The same amounts of protein samples were added into per lane and separated by 10% sodium dodecyl sulfate polyacrylamide gels electrophoresis (SDS-PAGE). Subsequently, the protein was transferred to PVDF membrane (Millipore, USA), and the following steps were performed such as blocking, primary antibodies incubation against Bcl-2 (1:3000, Cell Signaling Technology), Bax (1:3000, Cell Signaling Technology), and GAPDH (1:3000, Proteintech) overnight at 4 °C, and the secondary antibody incubation for 2 h at room temperature according to species of primary antibodies. Finally, the expression levels of proteins were identified using enhanced chemiluminescence system (ECL) (NCM Biotech, China) via gel imaging system (Bio-Rad, USA).

### Cell apoptosis assay

Annexin V FITC/PI apoptosis Kit (Multisciences, China) was applied to identify cell apoptosis. The cells were planted into six-well plates and cultivated at 37 °C for 24 h before the experiment. The cells were given the same treatment as in Western blot analysis. Subsequently, the harvested cells were incubated with FITC-Annexin V and PI for 5 min at room temperature. Finally, cells apoptosis assay was conducted via flow cytometry.

### Wound-healing assay

The wound healing assay was utilized to evaluate migration ability of tumor cell. Upon forming confluent monolayer in 6-well plate, the cells were treated with Apt-NS, DOX (2 μM), Apt-NS-DOX (equivalent DOX concentration) for 24 h, with setting the blank control group. Subsequently, the cells were scratched utilizing a sterile plastic tip. The migration ability was evaluated by comparing the width of cell scratch at different time points (24 and 48 h).

### Transwell assay

Transwell assay was conducted to evaluate the ability of cells migration and invasion. Briefly, the cells were treated with the same way as in wound-healing assay. Then the cells (5 × 10^4^ cells/well) resuspended with 200 μL serum-free medium were added into the upper chamber of the insert (Millipore) equipped with and without matrigel (BD Bioscience) for assessing the ability of cell invasion and migration, respectively. Then, 500 μL medium containing 10% FBS was supplied in the lower well. After incubation for 24 h at 37 °C, the cells on the upper surface were eliminated and the cells located on the lower side of the membrane were subjected to fixation with 4% paraformaldehyde (30 min) and staining with crystal violet (15 min), and then counted in three randomly chosen fields of view for further statistical analysis.

### Xenograft tumor model in nude mice

Animal experiments were conducted with an approval by ethics committee of the First Affiliated Hospital of Wenzhou Medical University. BALB/c nude mice (female, about 20 g, 4 − 6 week old) were provided by the animal experimental center of the First Affiliated Hospital of Wenzhou Medical University. All animals were housed and bred in specific pathogen-free (SPF) environment. To establish xenograft tumor model, the subcutaneous injection of A549 cells (5 × 10^6^ cells/100 μL PBS) was conducted in the lateral of right axilla.

### In vivo antitumor experiments

The xenograft tumor mice (when the A549 tumor diameter reached about 5 mm) were randomly divided into four groups (n = 5), and separately treated with PBS, DOX, Lib-NS-DOX, and Apt-NS-DOX (S6-NS-DOX) by tail vein injections, wherein the equivalent concentration of DOX was 2 mg/kg. The first injection was considered as day 0, and the intravenous injection was conducted every 3 days for 18 days. The measurements such as tumor volume and body weight were simultaneously performed. Tumor volume was calculated based on the following formula: tumor volume = length × (width)^2^/2. After treatment, the major organs and tumor tissues were harvested for histologic examination.

### Tissues staining

The major organs and tumor tissues were subjected to fixation with 4% paraformaldehyde (24 h) and paraffin embed, and then sectioned into slices (4 μm thickness). The tissue sections were conducted hematoxylin and eosin (H&E) staining according to standard protocols. For evaluating tumor cells proliferation, tissue samples were immunostained with Ki67 antibody according to standard protocols. The images of HE and immunohistochemical staining were observed using a light microscope (Nikon, Japan). Ki67-positive cells was counted for statistical analysis. For evaluating cell apoptosis in tumor tissue, terminal deoxynucleotidyl transferase dUTP nick end labeling (TUNEL) staining was performed utilizing One-Step TUNEL Apoptosis Kit (Elabscience, China). The green fluorescence of TUNEL-positive cells was detected using fluorescence microscopy (Nikon, Japan), followed by statistical analysis.

### Statistical analysis

The data of cell culture experiments are acquired from three independent experiments. Quantitative data were presented as mean ± standard deviation (SD). The difference between two groups was analyzed using Student's t test or Wilcoxon rank sum test. The comparison of multiple experimental groups was performed using one-way analysis of variance (ANOVA). Statistical analysis was accomplished via GraphPad Prism 8, and *P* < 0.05 was considered statistically significant.

## Results and discussion

### Preparation and characterization of DNA nanostructure

Currently, self-assembly of single strands DNA is an important synthetic strategy, which has been widely applied for the construction of DNA nanostructure [29–33]. In this study, we developed a A549 cell-targeting drug delivery system (Apt-NS) based on DNA nanostructure, which was synthesized by one-step assembly process (Scheme 1). Specifically, Apt-NS was assembled by three ssDNA, including S6 (aptamer), PP (palindrome probe), and Linker, of which PP and Linker contributed to structure assembly, and S6 was connected with PP using the complementary sticky end, realizing the tumor-targeting capacity of DNA nanostructure. The detail self-assembly process from single-strand DNA to a structural unit was displayed in Additional file 1: Scheme S1.

**Fig. 1:**
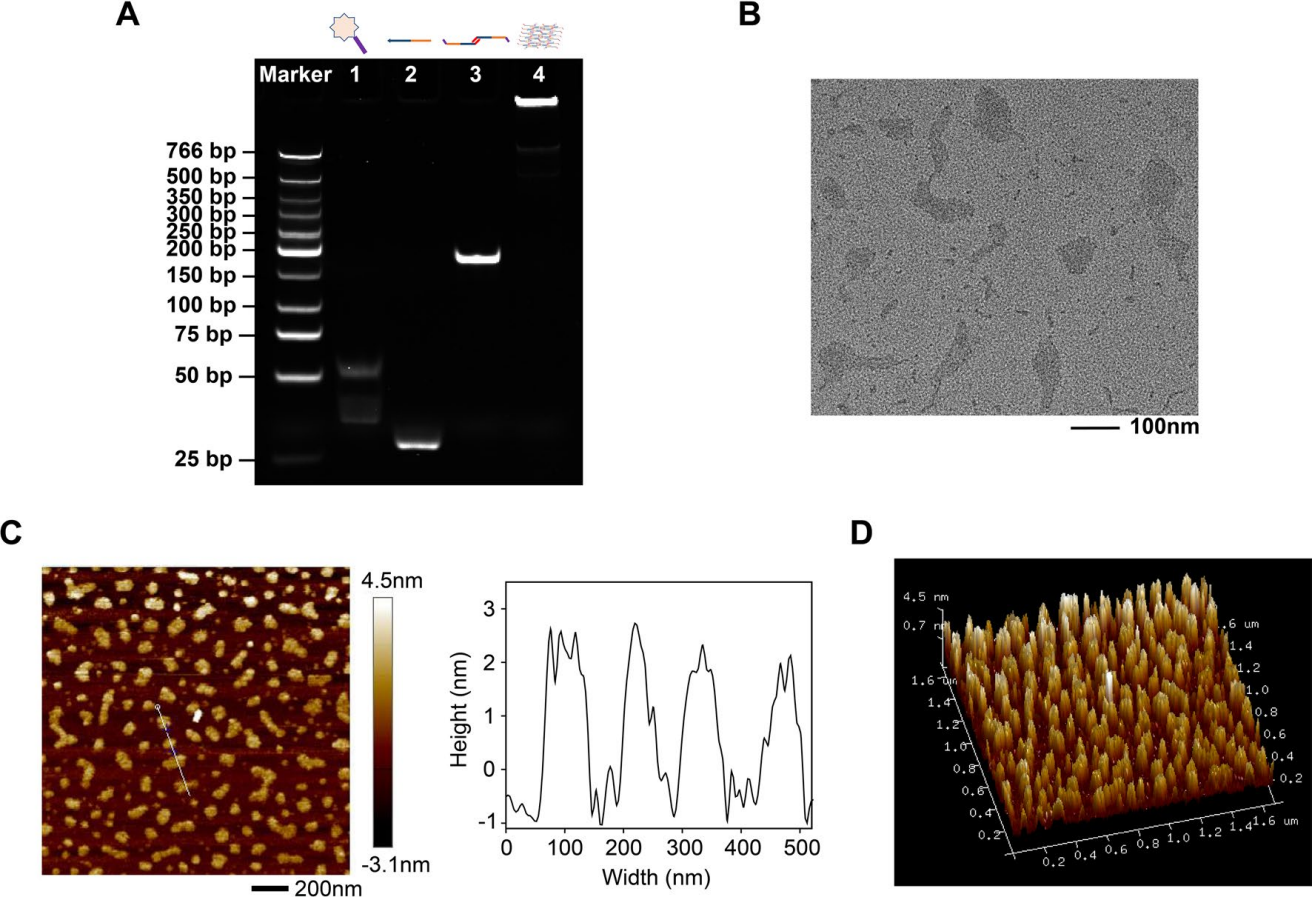
Synthesis and characterization of Apt-NS. **A** Gel electrophoresis analysis of 8% PAGE. Lane 1, 2 and 3 indicate S6 aptamer, Linker, and PP dimer, respectively. Lane 4 indicates Apt-NS. **B** TEM image of Apt-NS. **C** AFM image of Apt-NS. **D** The 3D view of AFM image

In order to validate the feasibility of the design, 8% PAGE was first performed to identify the electrophoretic mobility. As shown in Fig. 1A, Lane 1 and 2 represented S6 (61 bases) and Linker (40 bases), respectively. Lane 3 represented the PP (69 bases) dimer formed by palindromic sequence containing 14 bases. Thus, the band in Lane 3 displayed a slower electrophoretic mobility than 69-base single-strand DNA. Lane 4 represented self-assembly of Apt-NS. We discovered that Lane 4 DNA sample appeared a rather slow electrophoretic mobility in 8% PAGE (almost blocking in the loading well), suggesting the formation of a larger structure. The results confirmed that Apt-NS could be successfully prepared, suggesting the assembly scheme was feasible. Taking advantage of self-assembly strategy, DNA nanostructure could be easily prepared in the absence of enzymatic reactions conditions.

To investigate the significant role of palindromic sequence in the assembly of Apt-NS, we designed CP (common probe) without palindromic sequence instead of PP (palindrome probe) and conducted the self-assembly under the same conditions. As shown in Additional file 1: Figure S1, Lane 3 represented CP (69 bases) without palindromic sequence. The electrophoretic mobility of Lane 3 in Additional file 1: Figure S1 was significantly faster than that of Lane 3 in Fig. 1A, inferring that CP dimer was not formed. Meanwhile, there was multiple bands present in Lane 4, and some bands appeared faster migrating, revealing that the assembly of DNA nanostructure was clearly affected in the absence of palindromic sequence. Thus, this result confirmed that the design of palindromic sequence was essential for the assembly of Apt-NS. Next, TEM image distinctly exhibited the formation of Apt-NS, of which the long diameter of Apt-NS was approximately 100 nm (Fig. 1B). Meanwhile, AFM image also confirmed the assembly of Apt-NS (Fig. 1C). The average length and width of Apt-NS were 101 ± 30 nm and 58 ± 8 nm, respectively (Additional file 1: Figure S2). The AFM result was broadly consistent with the size of Apt-NS observed in TEM image. According to the 3D view of AFM image (Fig. 1D), Apt-NS displayed a planar structure due to the lower height (about 2–3 nm).

### Stability analysis of Apt-NS

Stability is a key consideration in the application of targeted drug delivery system. Therefore, it is essential to verify the stability of Apt-NS. Owing to the interference of nanomaterial functions by various serum proteins in FBS [34, 35], we first compared the stability of ssDNA (Linker and PP) and Apt-NS in a 10% FBS environment. The results indicated that there was still a relatively sharp band in 8% PAGE, when Apt-NS was incubated with 10% FBS for 2 h, 6 h, 12 h, and 24 h. In contrast, the two ssDNA Linker and PP was rapidly degraded after the same treatment. Quantitative analysis further showed that the complete structure of Apt-NS still maintained more than 60% after 24 h (Additional file 1: Figure S3A). These results demonstrated that Apt-NS presented a good stability in serum. Since DNA nanostructure could be disassembled by DNase I [36], DNase I stability of Apt-NS was then explored. Apt-NS was treated with DNase I for 2 h, 6 h, 12 h, and 24 h, and the structural integrity of Apt-NS also maintained about 60% after 24 h (Additional file 1: Figure S3B). The comprehensive results of stability analyses confirmed that Apt-NS exhibited much better stability, which provided guarantees for DNA nanostructure in efficient drug delivery and cell internalization. In addition, sufficient stability improved the blood circulation time of Apt-NS, laying down the foundation for the application in vivo.

### Cell-specific uptake of Apt-NS

Cell-specific uptake is an essential precondition for antitumor therapy based on targeted drug delivery system. To investigate cell uptake abilities, Cy5 was labeled for tracking and positioning the location of DNA nanostructure [37]. Flow cytometry and CLSM were utilized to assess the targeting ability of Apt-NS. We first investigated cell recognition ability of the different concentrations (200 nM, 500 nM, 800 nM, 1 μM, 2 μM) of S6 aptamer by A549 cells using flow cytometry. The results revealed that as the increase of S6 aptamer concentration, the fluorescence intensity gradually increased and reached the maximum when S6 concentration was 800 nM (Additional file 1: Figure S4A). Meanwhile, CLSM images displayed the similar results that A549 cells emitted the brightest fluorescence when S6 concentration reached to 800 nM (Additional file 1: Figure S4B). According to these results, we confirmed that 800 nM was the optimal concentration for S6 aptamer recognizing the target A549 cells. Thus, S6 aptamer concentration (800 nM) was used for the subsequent experiments.

**Fig. 2:**
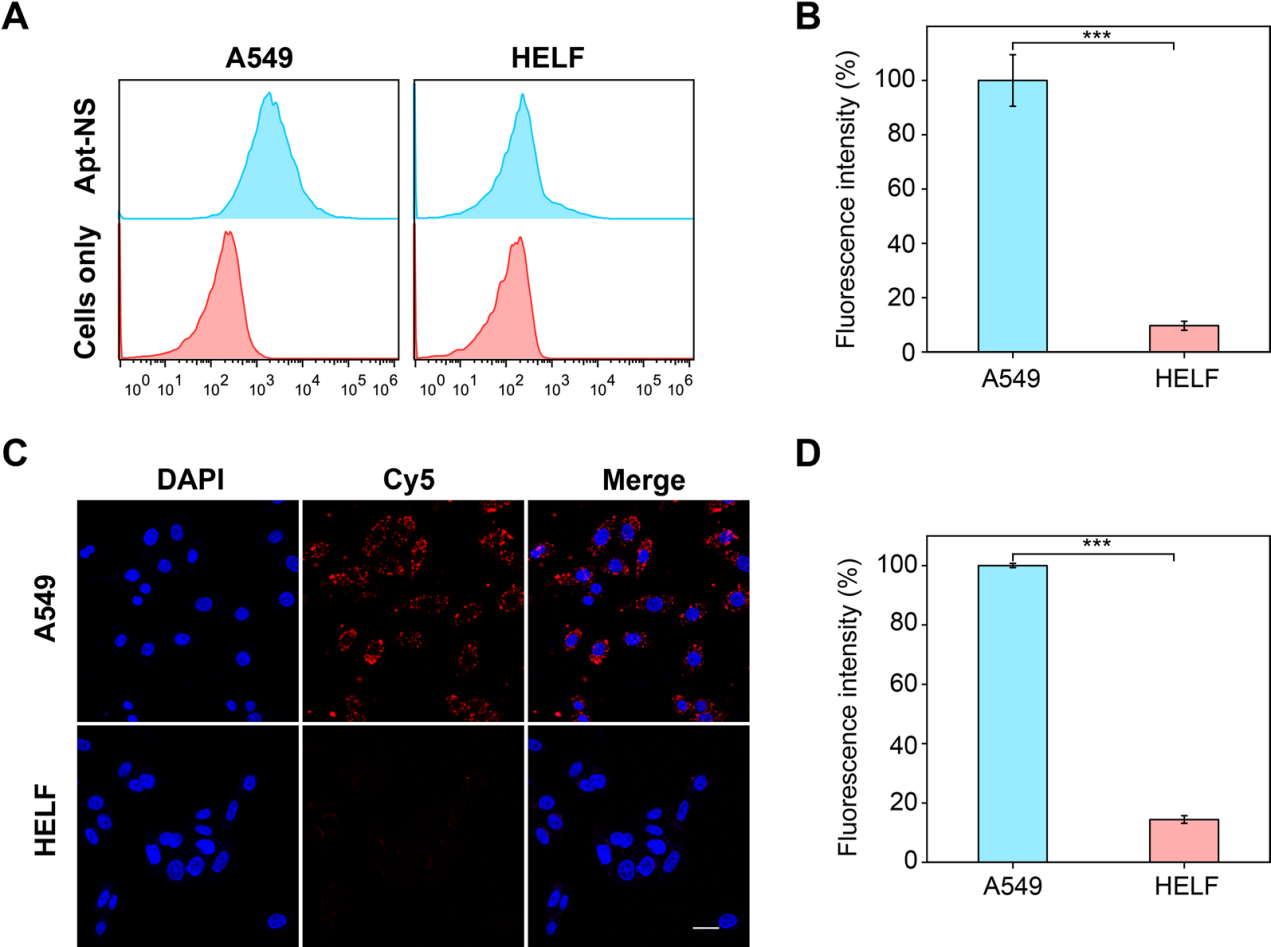
Efficient cellular recognition of Apt-NS to A549 cells. **A** Flow cytometry analysis of A549 and HELF cells for the internalization of Apt-NS. **B** Quantitative analysis of the mean fluorescence intensity of each group in (**A**) (n = 3). 10,000 events were recorded for each sample assay. Statistical analysis: ***P < 0.001. (C) CLSM images of A549 and HELF cells treated with Apt-NS (red). Nucleus are stained blue. Scale bar is 25 μm. **D** Quantitative analysis of the mean fluorescence intensity of each group in (**C**) (n = 3). Statistical analysis: ***P < 0.001

As shown in Fig. 2A, flow cytometry results indicated that obvious fluorescence signal change was appeared in A549 cells treated with Cy5-labeled Apt-NS, compared to blank control group (cells only). Quantitative results also displayed a high fluorescence intensity in A549 cells (Fig. 2B). In contrast, no obvious fluorescence signal change was observed in HELF cells underwent the same treatment. In addition, the similar results were observed in confocal fluorescence imaging. After the same treatment, A549 cells exhibited a stronger fluorescence due to significantly increased cells internalization, while a slight fluorescence was emitted from HELF cells (Fig. 2C). Quantitative analysis results revealed that the fluorescence signals of A549 cells group presented approximate sevenfold stronger than those of HELF cells group, demonstrating the remarkably increased cellular uptake efficiency of Apt-NS by A549 cells (Fig. 2D). These results derived from flow cytometry and CLSM consistently confirmed that the specific targeting ability of Apt-NS to A549 cells.

### Internalization pathway and intracellular colocalization of Apt-NS

**Fig. 3:**
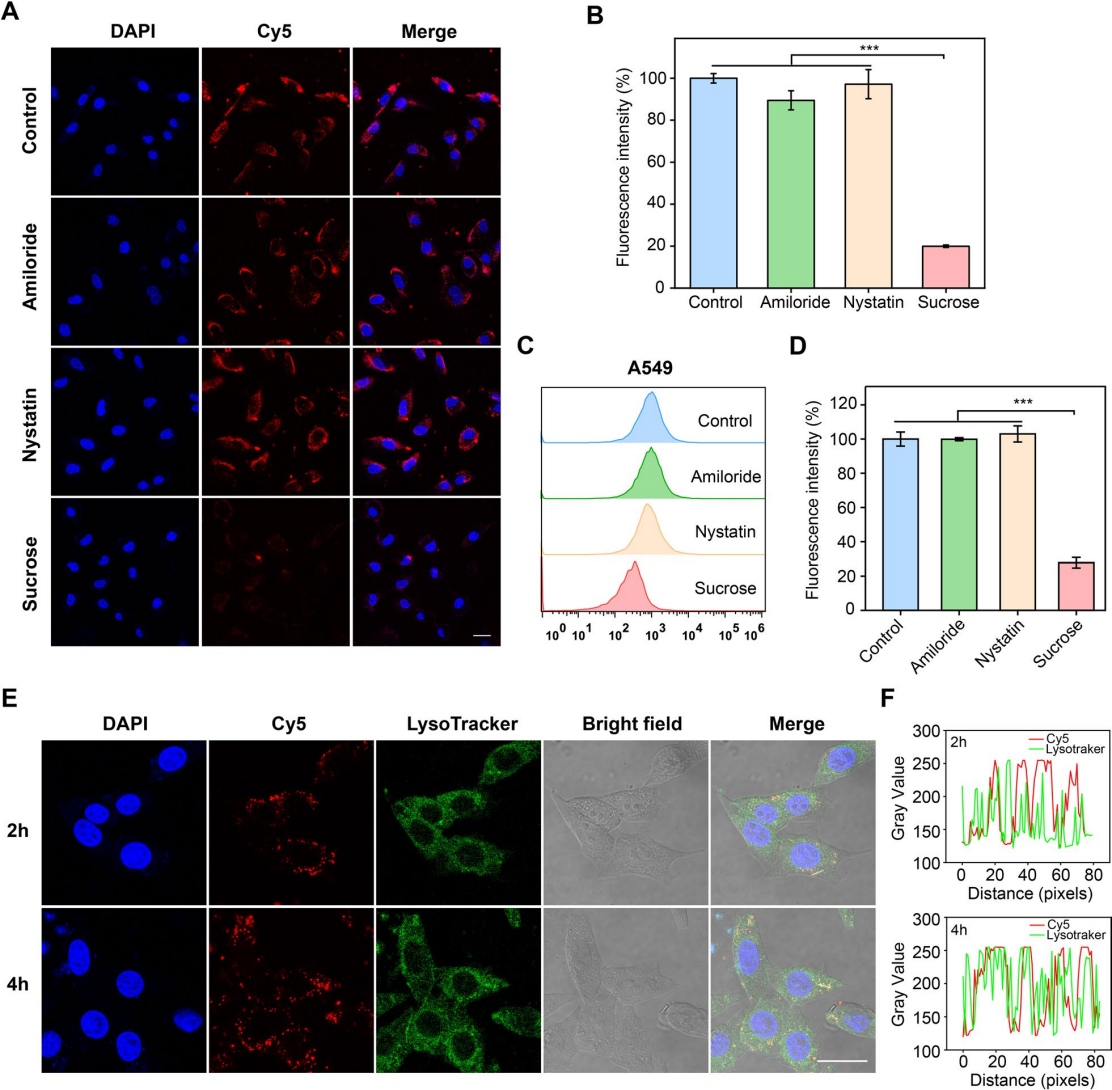
Cellular uptake mechanism and intracellular colocalization of Apt-NS. **A** Confocal images showing the influence on Apt-NS uptake in A549 cells treated by different endocytic inhibitors. **B** Quantitative analysis of the mean fluorescence intensity of each group in (**A**) (n = 3). Statistical analysis: ***P < 0.001. **C** Flow cytometric assay showing the influence on Apt-NS uptake in A549 cells treated by different endocytic inhibitors. **D** Quantitative analysis of the mean fluorescence intensity of each group in (**C**) (n = 3). Statistical analysis: ***P < 0.001. **E** Confocal images showing colocalization of Apt-NS with lysosome. **F** The fluorescence intensity of LysoTracker (green) and Cy5-labeled Apt-NS (red) at the same site of marker positions (white line) in the overlay image of (**E**). Scale bar is 25 μm

To further investigate the internalization pathway of Apt-NS, three different endocytosis inhibitors such as sucrose [38], amiloride [39], and nystatin [40] were selected in this experiment, which specifically blocked clathrin, micropinocytosis, and caveolin-mediated internalization pathways, respectively. We first performed the confocal image analysis, and the results indicated that the cellular internalization of Apt-NS was obviously suppressed in sucrose treatment group, while amiloride and nystatin had no significant inhibitory effects on cellular internalization of Apt-NS (Fig. 3A, B). Furthermore, similar results were observed in flow cytometry. There was obvious fluorescence signal decreased in A549 cells treated with sucrose, compared to amiloride and nystatin treatment groups (Fig. 3C, D). Our combined results confirmed that the internalization pathway of Apt-NS by A549 cells mainly depended on clathrin-mediated endocytosis. Previous studies have found that nanocarriers with size about 100 nm generally entry into cells relying on clathrin-mediated endocytosis [39]. In our study, the size of Apt-NS also reached 100 nm, and the results of Apt-NS internalization pathway analysis were consistent with those reported in literature.

Previous studies have reported that DNA nanostructure can be degraded by lysosomes, with providing essential hydrolases for cargo release [41–44]. Therefore, the subcellular localization of Apt-NS in A549 cells was assessed using CLSM, where Apt-NS was labeled by Cy5 (red) and lysosomes were stained by LysoTracker (green). At 2 h, the confocal images displayed that there was fewer overlap fluorescence between the Cy5 signal and LysoTracker signal. However, at 4 h, enhanced overlapped fluorescent was observed, suggesting the number of Apt-NS entered into lysosomes was gradually increased (Fig. 3E, F). These results revealed that Apt-NS was internalized into cells by clathrin-mediated endocytosis, and then transferred to lysosome by the endosome.

### Targeted drug delivery of Apt-NS-DOX

**Fig. 4:**
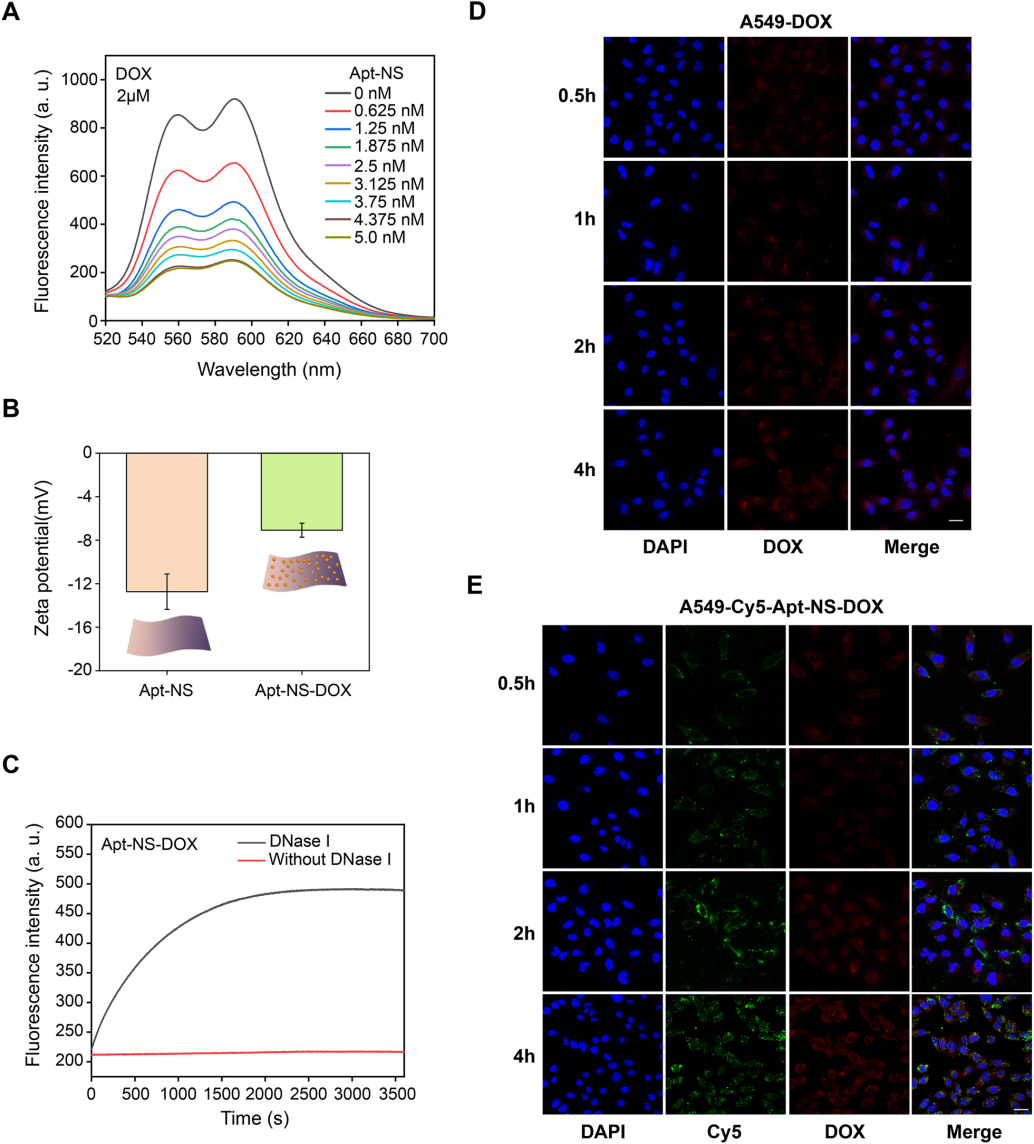
Drug loading and release properties of Apt-NS and targeted drug delivery by Apt-NS-DOX in A549 cells. **A** Fluorescence intensity alterations of DOX as the increase of Apt-NS. **B** Zeta potentials assay of Apt-NS and Apt-NS-DOX. **C** Fluorescence signals detection of DOX released from Apt-NS-DOX. Black line indicated the changing trends of fluorescence signals of Apt-NS-DOX treated with DNase I enzyme. Red line indicated the changing trends of fluorescence signals of Apt-NS-DOX treated with the buffer without adding DNase I enzyme. **D** Confocal images showing the time-dependent cellular uptake of free DOX by A549 cells. Red fluorescence represented the free DOX. **E** Confocal images showing the time-dependent cellular uptake of Apt-NS-DOX by A549 cells. Red fluorescence represented the DOX released from Apt-NS-DOX; green fluorescence represented Cy5 labeled nanomaterials. Scale bar is 25 μm

According to the analyses and findings above, Apt-NS was successfully prepared and its tumor-targeting feature was further confirmed, which could be applied as deliver carrier to transport the chemotherapeutic drug into target tumor cell, exerting antitumor effects. It is well known that DNA nanostructure can be loaded with anthracyclines owing to the rich of GC base pair [45]. DOX, a widely used anthracycline anticancer agent, can insert into double-stranded DNAs, inducing the quenching of drug fluorescence due to fluorescence resonance energy transfer (FRET) [46–48]. Thus, DOX loading and release of DNA nanostructure can be evaluated via the changes of fluorescence. First, it was observed that with the increase of Apt-NS (from 0 to 5.0 nM), DOX fluorescence decreased successively (Fig. 4A). And when the concentration of Apt-NS was increased to 4.375 nM, the fluorescence was no longer decreased significantly, even if Apt-NS was further increased. This result demonstrated that almost all the DOX molecules were combined with Apt-NS.

The drug-carrying capacity of DNA nanostructures can be assessed by the amount of DOX loaded by a single nanoparticle [48]. According to the changes of spectra, when the concentration of Apt-NS was 4.375 nM (the molar ratio of Apt-NS to DOX reached to 1:457), the fluorescence quenching efficiency reached the maximum. This result indicated that Apt-NS presented a quite high drug loading capacity. Furthermore, as the adding of DOX, the zeta potential of Apt-NS emerged the decreased of negative charge, also suggesting the binding between Apt-NS and DOX (Fig. 4B), which was due to the negative charge of Apt-NS being partially neutralized by the positive charge of DOX. Additionally, DOX (10 µM) was incubated with Apt-NS at room temperature overnight, and the red precipitate (Apt-NS-DOX) could be obtained after centrifugation (Additional file 1: Figure S5). These results above confirmed that DOX could be successfully loaded onto Apt-NS, laying an important foundation for the applications for drug delivery. Subsequently, fluorescence recovery test was performed to explore whether DOX could be released from Apt-NS-DOX. As shown in Fig. 4C, the DOX fluorescence was gradually increased and finally reached a plateau under the action of DNase I enzyme (20 U/ml). In contrast, there was no significant fluorescence increasing in the samples without DNase I treatment. This result confirmed that DOX could be successfully released from Apt-NS-DOX, guaranteeing the antitumor effects after the degradation of Apt-NS-DOX in target cells.

In order to investigate the cancer-targeted delivery of Apt-NS-DOX, we observed the cellular internalization of Cy5 labeled Apt-NS-DOX in A549 and HELF cells, where Cy5-Apt-NS-DOX contained two colors (Cy5, green fluorescence (F-green); DOX, red fluorescence (F-red)). For target A549 cells, confocal images exhibited that F-green and F-red were all present and gradually increased over time (Fig. 4E). Notably, F-green predated the appearance of F-red, which was consistent with the process that DOX was gradually released from tumor cell internalized Apt-NS-DOX and F-red of DOX was gradually increased, because F-red of DOX was quenched in the status of Apt-NS-DOX. However, there were no obvious F-green and F-red in nontarget HELF cells treated with Cy5-Apt-NS-DOX (Additional file 1: Figure S6B). In contrast, the fluorescence of free DOX (F-red) was all detected in A549 and HELF cells without cell selectivity (A549 cells in Fig. 4D and HELF cells in Figure S6A). Based on these data, we confirmed that Apt-NS-DOX performed excellent tumor targeted drug delivery and intracellular release capabilities in the anticancer therapy.

### Disassembly of Apt-NS-DOX in cells exerting antitumor effect

**Fig. 5:**
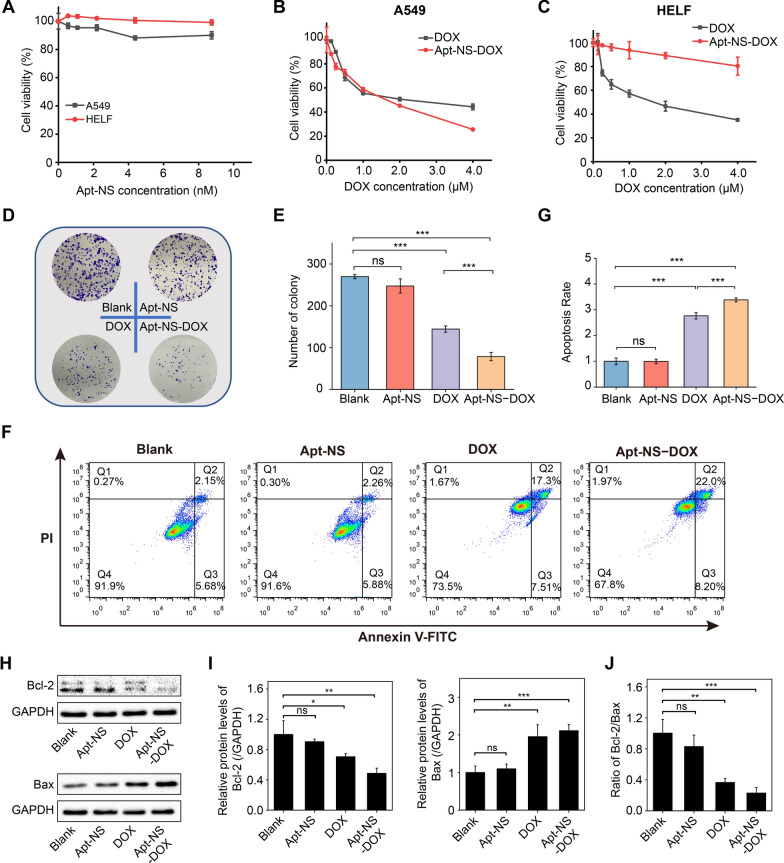
Antitumor effects of Apt-NS-DOX on cell proliferation and apoptosis. **A** Cell viability analysis of both A549 and HELF cells treated with Apt-NS. **B** Cell viability analysis of A549 cells treated with free DOX and Apt-NS-DOX, respectively. **C** Cell viability analysis of HELF cells treated with free DOX and Apt-NS-DOX, respectively. **D** Colony formation assay of A549 cells treated with Apt-NS, DOX, and Apt-NS-DOX, respectively. **E** Quantitative analysis of the number of colonies of each group in (D). **F** Flow apoptosis assay of A549 cells treated with Apt-NS, DOX, and Apt-NS-DOX, respectively (Q1 means dead cells; Q2 means late apoptotic cells; Q3 means early apoptotic cells; Q4 means normal cells). **G** Quantitative analysis of cell apoptosis rate of each group in (**F**). **H** Western blot analysis displaying the expression levels of Bcl-2 and Bax proteins in A549 cells treated with Apt-NS, DOX, and Apt-NS-DOX, respectively. **I** Quantitative analysis of Bcl-2 and Bax proteins expression of each group in (**H**). GAPDH was used as an internal control. **J** The ratio of Bcl-2/Bax analysis in A549 cells treated with Apt-NS, DOX, and Apt-NS-DOX, respectively. Statistical analysis: *P < 0.05, **P < 0.01, ***P < 0.001 and ns, no significance

The nanoparticles (Apt-NS-DOX) were internalized into target cells by cell-specific aptamer S6, and then DOX would be released in lysosomes after the degradation of Apt-NS-DOX. The DOX could enter into nucleus and induced DNA damage exerting antitumor effect (Additional file 1: Figure S7). To further explore the influence of A549 and HELF cells treated with Apt-NS, DOX, and Apt-NS-DOX, the cell viability analysis was performed. We first evaluated the cell toxicity of Apt-NS (unloading drug) in the two cell lines. As shown in Fig. 5A, Apt-NS appeared very low toxicity in both target A549 cells and nontarget HELF cells, demonstrating that Apt-NS exhibited good biocompatibility as the ideal vehicles for drug delivery, which was consistent with that of DNA nanostructures in previous reports [49, 50]. Moreover, the essence of Apt-NS is DNA, which can be degraded in the body with no toxic residue. This feature is distinguished from other traditional nanomaterials, including gold nanoparticle, which can easily lead to the accumulation of heavy metals and harm the normal cells and organs. Furthermore, cytotoxic effects of DOX and Apt-NS-DOX on the two types of cells were investigated via the same procedure, respectively. As shown in Fig. 5B, C, free DOX exhibited dose-dependent cytotoxicity to both A549 and HELF cells. In contrast, Apt-NS-DOX mainly exerted obvious antitumor effects on target A549 cells, while a relatively low toxicity was observed in nontarget HELF cells. The high cytotoxicity of Apt-NS-DOX in A549 cells was consistent with the trend of higher cellular uptake of nanomaterials by A549 cells. Besides, Apt-NS-DOX exhibited remarkable dose-dependent inhibitory effects on tumor cell growth. These comparative results from target and nontarget cells revealed the satisfactory selective cells killing owing to the precise delivery of chemotherapeutic drug via Apt-NS. In addition, colony formation assay also revealed that Apt-NS-DOX exerted the most obvious inhibitory effects on colony formation ability of tumor cells compared to other groups, such as Blank, Apt-NS, and DOX (Fig. 5D, E). Based on all results shown above, we determined that Apt-NS-DOX performed significant effects on inhibiting cancer cells proliferation.

DOX, as one of conventional chemotherapy drugs, has been reported to induce cell apoptosis [51, 52]. The cell apoptosis caused by different formulations in target cells and nontarget cells were further evaluated using flow cytometry. The apoptosis rates of A549 cells were detected after the different treatment of Blank, Apt-NS, DOX, and Apt-NS-DOX, respectively (Fig. 5F, G). Both Blank control and Apt-NS groups played a negligible role in A549 cell apoptosis. DOX, particularly Apt-NS-DOX, remarkably promoted A549 cell apoptosis compared to Blank/Apt-NS groups. Additionally, the percentage of apoptotic cells increased from 24.81% (DOX) to 30.2% (Apt-NS-DOX), suggesting that effective intracellular release of DOX exerted stronger apoptotic effects on target A549 cells. In contrast, as shown in Additional file 1: Figure S8A and B, free DOX could indiscriminately enter into HELF cell resulting in obvious cell apoptosis, while Apt-NS-DOX induced inconspicuous cell apoptosis due to the undesirable cell recognition, compared to Blank control/Apt-NS groups. These results from cell apoptosis assay were consistent with cell viability assay, demonstrating the excellent antitumor ability of Apt-NS-DOX. Next, we further investigated the potential mechanism of Apt-NS-DOX inducing cell apoptosis. The expression level of apoptosis-related proteins, such as Bcl-2 (anti-apoptosis protein) and Bax (proapoptotic protein) were detected by Western blot analysis. Apt-NS negligibly affected the expression level of Bcl-2 and Bax compared to the Blank control group. There appeared down-regulation of Bcl-2 protein and up-regulation of Bax protein in DOX and Apt-NS-DOX groups, wherein Apt-NS-DOX showed more obvious alterations of apoptosis-related protein expression (Fig. 5H, I). Additionally, the ratio of Bcl-2/Bax can be considered as a vital determinant of cell survival or death when apoptosis is triggered [53]. We discovered that the ratio of Bcl-2/Bax was remarkably decreased by Apt-NS-DOX (Fig. 5J). Based on these experimental results mentioned above, Apt-NS-DOX performed a crucial role in inducing cell apoptosis by intervening the expression level of apoptosis-related proteins.

**Fig. 6:**
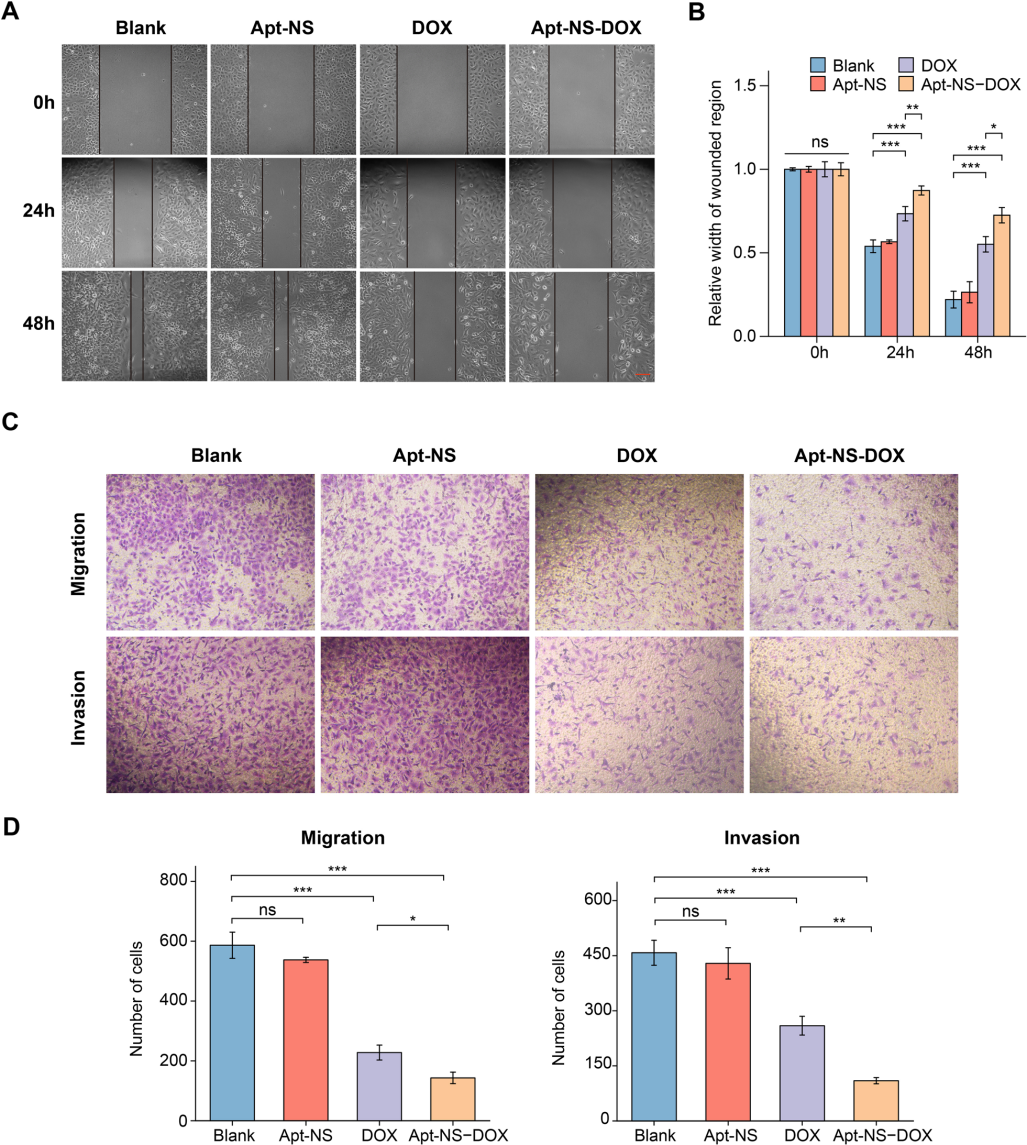
Antitumor effects of Apt-NS-DOX on cell metastasis. **A** The wound healing assay of A549 cells treated with Apt-NS, DOX, and Apt-NS-DOX, respectively. **B** Quantitative analysis of cell migration of each group in (**A**). **C** Transwell assay of A549 cells treated with Apt-NS, DOX, and Apt-NS-DOX, respectively. **D** Quantitative analysis of cell migration and invasion of each group in (**C**). Statistical analysis: *P < 0.05, **P < 0.01, ***P < 0.001 and ns, no significance

Some studies have uncovered that DOX loading nanoparticles can efficiently control tumor progression by attenuating the tumor cell metastasis [54, 55]. Thus, the inhibitory effect of Apt-NS-DOX on A549 cells metastasis was investigated using wound healing and transwell assay. The wound healing assay first indicated that the migration ability of A549 cells was suppressed by DOX/Apt-NS-DOX, and the more significant inhibition effects could be observed in Apt-NS-DOX group (Fig. 6A, B). Furthermore, transwell assay revealed that DOX/Apt-NS-DOX could significantly attenuate the migration and invasion ability of tumor cells, while Apt-NS-DOX had a stronger effect (Fig. 6C, D). These results confirmed that Apt-NS-DOX had a significant antitumor metastasis ability, demonstrating many advantages of nanomaterials to the efficient and sustainable inhibitory influences on tumor cells.

### *Disassembly of Apt-NS-DOX *in vivo* exerting antitumor effect*

Biodistribution and tumor targeting imaging were first investigated utilizing the xenograft tumor model in nude mice. Cy5-labeled Apt-NS and Lib-NS were intravenously injected into subcutaneous tumor-bearing mice, respectively. For organ imaging of nanomaterial biodistribution, the fluorescence signals of Cy5-labeled Apt-NS and Lib-NS mainly emerged in liver and kidney, and a few in lung, while no obvious fluorescence signal was observed in heart and spleen. A possible cause of the obvious fluorescence signal appeared in liver and kidney was their scavenging effects on nanomaterials [56, 57]. For tumor-targeting imaging, we found that the fluorescence signal of Cy5-labeled Apt-NS was significantly apparent in tumor tissue, while that of Cy5-labeled Lib-NS was not obvious. (Additional file 1: Figure S9A). Meanwhile, quantification analysis results of average fluorescence intensity of regions of interest (ROI) also confirmed that Apt-NS significantly gathered in tumor tissue as compared to Lib-NS (Additional file 1: Figure S9B). These results demonstrated that aptamer-modified DNA nanostructure (Apt-NS) could specifically recognize the tumor tissue.

We further evaluated the biocompatibility of nanomaterials using the hemolysis assay. As shown in Additional file 1: Figure S10, there presented an obvious occurrence of hemolysis in the positive control group (H_2_O treatment). In the negative control group (PBS treatment), the hemolysis rate was lower than 1%, without no obvious hemolysis. For nanomaterials treatment group (S6-NS-DOX treatment with concentrations ranging from 2.5 to 40 μg/mL), the hemolysis rates presented lower than 4%, suggesting that there had negligible influences of S6-NS-DOX on red blood cells (RBCs), with a better biocompatibility.

**Fig. 7:**
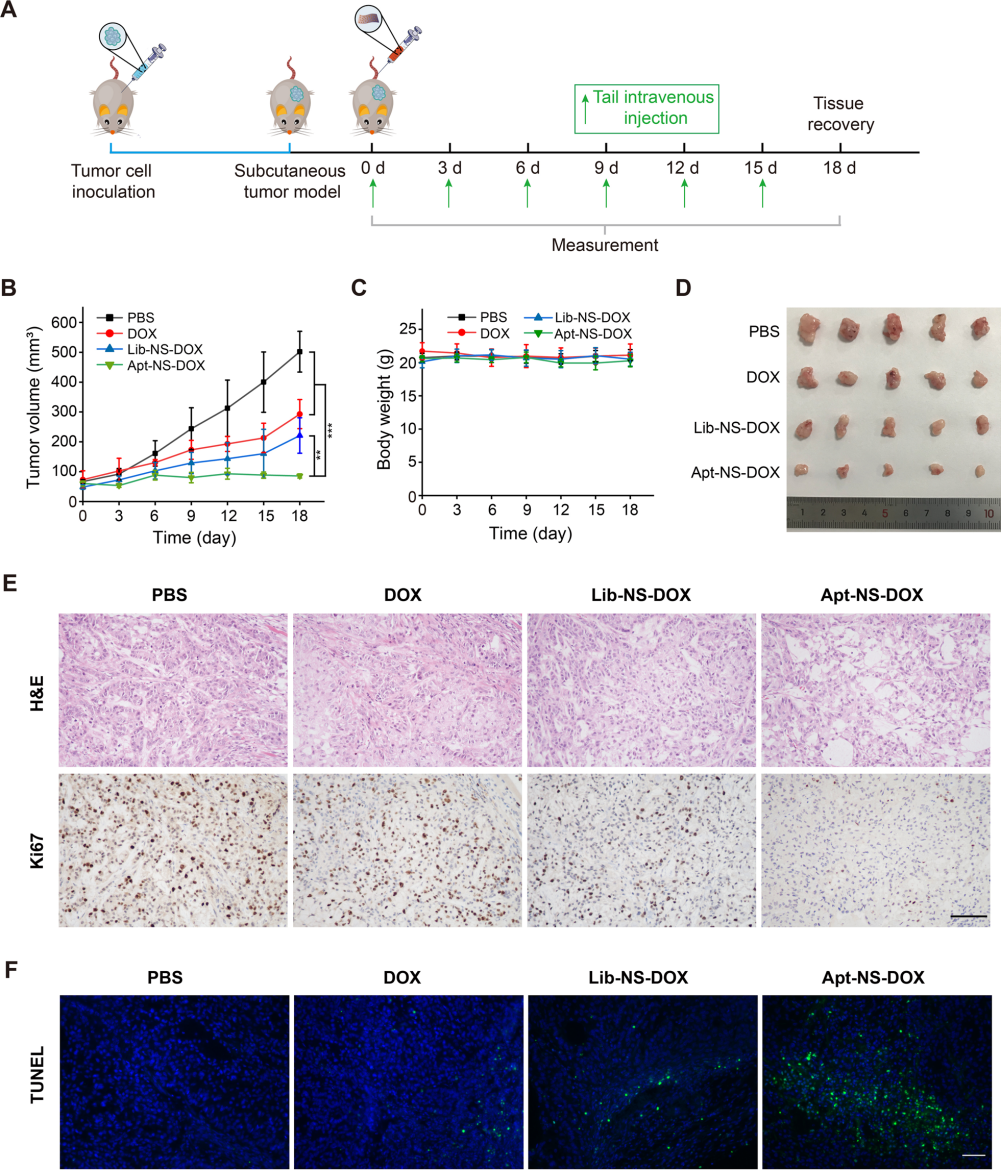
Antitumor effects of free DOX, Lib-NS-DOX, and Apt-NS-DOX in xenograft tumor model. **A** Schematic diagram of the arrangement for the therapeutic experiments. After the treatment of free DOX, Lib-NS-DOX, and Apt-NS-DOX, the changes of **B** tumor volume and **C** body weight were measured (n = 5). Statistical analysis: **P < 0.01, ***P < 0.001. **D** The digital images of tumors dissected from the mice at the end of experiment (n = 5). **E** H&E and Ki67 IHC staining of tumor tissues in the free DOX, Lib-NS-DOX, and Apt-NS-DOX treated groups. **F** TUNEL staining of tumor tissues in the free DOX, Lib-NS-DOX, and Apt-NS-DOX treated groups. Green represented apoptotic cells and blue represented nuclei. Scale bars are 50 µm

Next, in vivo antitumor properties of Apt-NS-DOX was explored via tail vein injection of Apt-NS-DOX and control agents (PBS, DOX, and Lib-NS-DOX) into subcutaneous tumor-bearing mice (Fig. 7A). Compared with control groups (PBS, DOX, and Lib-NS-DOX), Apt-NS-DOX exerted the remarkably inhibitory effects on tumor growth, revealing the significant antitumor efficacy (Fig. 7B). The antitumor efficacy of Apt-NS-DOX was more enhanced than that of free DOX, and Lib-NS-DOX, which might due to that the targeting and high efficiency delivery properties of Apt-NS. Meanwhile, there were no evident changes in body weights in any group mice during the treatment period, revealing the relative safety of these injected reagents (Fig. 7C). The effective antitumor abilities of Apt-NS-DOX were observed in the photographs of the resected tumors in each group at the end of treatment (Fig. 7D). DOX in different forms (DOX, Lib-NS-DOX and Apt-NS-DOX) exhibited varying degrees of inhibitory effects on tumor growth based on xenograft tumor model. Notably, the antitumor ability of Apt-NS-DOX was superior to DOX and Lib-NS-DOX, which is largely due to the tumor-targeting and efficient delivery capability of Apt-NS enhancing the antitumor effects.

**Fig. 8:**
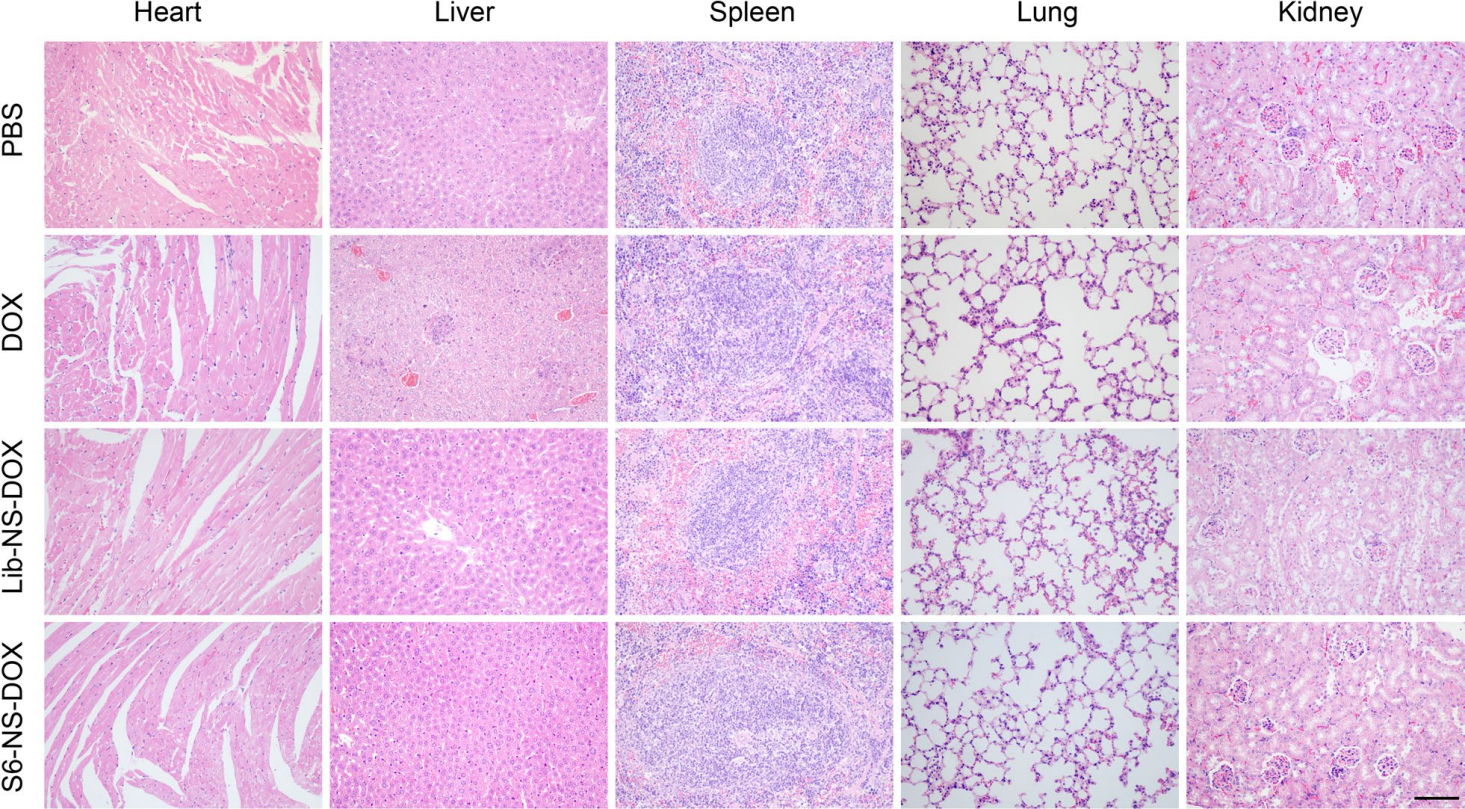
H&E staining of major organs (heart, liver, spleen, lung and kidney) showing the biosafety of different drug formulations. Scale bar is 50 μm

Biosafety and antitumor activity of Apt-NS-DOX were further evaluated using H&E, Ki67 antibody immunohistochemistry (IHC), and TUNEL staining methods. For biosafety analysis, H&E staining results of the major organs in each group indicated that no significant tissue necrosis occurred in these organs, such as heart, liver, spleen, lung, and kidney (Fig. 8). These results demonstrated that Apt-NS-DOX had good biosafety, with no organs toxicity. For antitumor ability analysis, H&E staining results of tumor tissues displayed that the nest tumor cells were significantly decreased in Apt-NS-DOX group. Furthermore, Ki67 staining was utilized to assess the proliferative activity of tumor cells [58]. The results revealed that the positive stained cells were also remarkably decreased in Apt-NS-DOX group, compared to other groups (PBS, DOX, and Lib-NS-DOX) (Fig. 7E and Additional file 1: Fig. S11). These results demonstrated that Apt-NS-DOX achieved the excellent ability on reducing tumor cell proliferation. In addition, TUNEL staining results suggested that there appeared a larger number of apoptotic cells (green) in Apt-NS-DOX group, compared to DOX and Lib-NS-DOX groups, indicating that Apt-NS-DOX had an enhanced ability on inducing cell apoptosis (Fig. 7F and Additional file 1: S12). Obviously, Ki67 and TUNEL staining results consistently confirmed that Apt-NS-DOX had tumor targeting and efficient intracellular drug delivery properties performing optimal antitumor effect in vivo. Combining the results above, Apt-NS-DOX could be identified as an excellent nanomaterial for cancer therapy.

## Conclusion

We successfully developed a novel targeting drug delivery system (Apt-NS-DOX) for NSCLC A549 cells, which could specifically deliver chemotherapeutic agents into tumor cells. This drug delivery system exhibited superior biocompatibility, profound suppression of tumor cell proliferation, invasion and migration, and also enhanced cell apoptosis. Meanwhile, Apt-NS-DOX could also efficiently transport DOX into tumor site of A549 tumor-bearing mice model by means of the characteristics of prolonged half-life and excellent biosafety. Apt-NS-DOX exerted enhanced antineoplastic efficacy without overt side effects compared to free drug, attributing to the precise delivery of antitumor drug. In summary, our study fully elucidated that DNA nanostructure could be considered as a promising nano-platform for targeted delivery of chemotherapeutic drugs with the advantages of high drug loading efficiency, the specific tumor accumulation, and less side effects. Based on this therapeutic strategy, such delivery platform can be further developed according to different aptamers and chemotherapy drugs, which will have excellent prospects for biomedical applications.

## Supplementary Information

**Additional file 1: Scheme S1.** The self-assembly process from single-strand DNA to a structural unit. The detail base sequences of three single-strand DNA, including S6 (aptamer), PP (palindrome probe), and Linker, are displayed in the pattern diagram. **Figure S1.** 8% PAGE image showing the influence of palindrome sequences on the synthesis of DNA nanostructure. CP (common probe) was instead of PP (palindrome probe). C-NS represented the synthetic products from self-assembly system in the absence of palindrome sequences. **Figure S2.** The calculation of Apt-NS sizes according to AFM images. Frequency distributions of the length and width of Apt-NS were assessed using NanoScope analysis software based on a method reported in the literature ^[1]^. The average length and width of Apt-NS were 101 ± 30 nm and 58 ± 8 nm, respectively. **Figure S3.** Stability assay of Apt-NS. (A) Serum stability assay of Apt-NS. PAGE analysis of samples such as Linker, PP, and Apt-NS (left panel). Quantitative assessment of Apt-NS serum stability based on gel electrophoresis images (right panel). (B) DNase I stability assay of Apt-NS. PAGE analysis of Apt-NS samples (upper panel). Quantitative assessment of Apt-NS DNase I stability based on gel electrophoresis images (lower panel). **Figure S4.** Assessment of cell recognition ability of the different concentrations of S6 aptamer on A549 cells. (A) Flow cytometric assay and (B) CLSM images showing the internalization of the different concentrations of S6 aptamer into A549 cells. Scale bar is 25 μm. **Figure S5.** Digital photos of DOX-loaded Apt-NS. (A, B) Photographs of free DOX (i) and DOX-loaded Apt-NS solution (ii) before and after centrifugation. **Figure S6.** Confocal images showing the time-dependent cellular uptake of DOX and Apt-NS-DOX by HELF cells. (A) Cellular uptake of free DOX by HELF cells. (B) Cellular uptake of Apt-NS-DOX by HELF cells. Red fluorescence indicated the DOX, green fluorescence indicated Cy5 labeled nanomaterials. Scale bar is 25 μm. **Figure S7.** Schematic illustration of the internalization and degradation of Apt-NS-DOX in A549 cells. Internalized Apt-NS-DOX was transferred to lysosomes, and then the DOX was released by lysosomes degradation. The released DOX could enter into nucleus and induced DNA damage. **Figure S8.** Apoptosis effects of Apt-NS-DOX on HELF cells. (A) Flow apoptosis assay of HELF cells treated with Apt-NS, DOX, and Apt-NS-DOX, respectively (Q1 means dead cells; Q2 means late apoptotic cells; Q3 means early apoptotic cells; Q4 means normal cells). (B) Quantitative analysis of cell apoptosis rate of each group in (A). Statistical analysis: ***P < 0.001 and ns, no significance. **Figure S9.** Nanomaterial biodistribution and tumor-targeting imaging in vivo. (A) fluorescence images of the major organs (heart, liver, spleen, lung and kidney) and tumor tissues. (B) Quantitative analysis of the average fluorescence intensities of each group in (A). Statistical analysis: **P < 0.01. **Figure S10.** Hemolysis assay for drug delivery system (Apt-NS-DOX). (+) represented a positive control (H_2_O) and (-) represented a negative control (PBS). Inset: the photographs of RBCs after incubation with the corresponding samples. The value presented as mean ± SD (n = 3). **Figure S11.** The calculation of Ki67-positive cells percentages according to the immunohistochemical staining of Ki67 in tumor tissues sections of different treatment groups. The value presented as mean ± SD (n = 5). Statistical analysis: ***P < 0.001. **Figure S12.** The calculation of apoptotic cell percentages according to the TUNEL staining of tumor tissues sections in different treatment groups. The value presented as mean ± SD (n = 3). Statistical analysis: ***P < 0.001. **Table S1.** Sequences of ssDNA designed in our experiments.

## Data Availability

All data and materials of this study can be obtained from the corresponding author under reasonable request.

## Abbreviations

AFM: Atomic force microscopy
CLSM: Confocal laser scanning microscopy
DOX: Doxorubicin
FBS: Fetal bovine serum
FRET: Fluorescence resonance energy transfer
HE: Hematoxylin and eosin
IHC: Immunohistochemistry
NAFN: Nucleic acid-based functional nanomaterials
NSCLC: Non-small cell lung cancer
PAGE: Polyacrylamide gel electrophoresis
ROI: Regions of interest
SDS-PAGE: Sodium dodecyl sulfate polyacrylamide gels electrophoresis
SELEX: Systematic Evolution of Ligands by Exponential enrichment
SPF: Specific pathogen-free
ssDNA: Single-strand DNA
TEM: Transmission electron microscopy
TUNEL: Terminal deoxynucleotidyl transferase dUTP nick end labeling
