# Supplementary material for: Self-assembly of DNA nanostructure containing cell-specific aptamer as a precise drug delivery system for cancer therapy in non-small cell lung cancer
Source: J Nanobiotechnology. 2022 Nov 19;20:486. doi: 10.1186/s12951-022-01701-5 (PMC9675138; doi:10.1186/s12951-022-01701-5)
Supplement: Supplementary file 1 — Additional file 1: Scheme S1. The self-assembly process from single-strand DNA to a structural unit. The detail base sequences of three single-strand DNA, including S6 (aptamer), PP (palindrome probe), and Linker, are displayed in the pattern diagram. Figure S1. 8% PAGE image showing the influence of palindrome sequences on the synthesis of DNA nanostructure. CP (common probe) was instead of PP (palindrome probe). C-NS represented the synthetic products from self-assembly system in the absence of palindrome sequences. Figure S2. The calculation of Apt-NS sizes according to AFM images. Frequency distributions of the length and width of Apt-NS were assessed using NanoScope analysis software based on a method reported in the literature [1]. The average length and width of Apt-NS were 101 ± 30 nm and 58 ± 8 nm, respectively. Figure S3. Stability assay of Apt-NS. (A) Serum stability assay of Apt-NS. PAGE analysis of samples such as Linker, PP, and Apt-NS (left panel). Quantitative assessment of Apt-NS serum stability based on gel electrophoresis images (right panel). (B) DNase I stability assay of Apt-NS. PAGE analysis of Apt-NS samples (upper panel). Quantitative assessment of Apt-NS DNase I stability based on gel electrophoresis images (lower panel). Figure S4. Assessment of cell recognition ability of the different concentrations of S6 aptamer on A549 cells. (A) Flow cytometric assay and (B) CLSM images showing the internalization of the different concentrations of S6 aptamer into A549 cells. Scale bar is 25 μm. Figure S5. Digital photos of DOX-loaded Apt-NS. (A, B) Photographs of free DOX (i) and DOX-loaded Apt-NS solution (ii) before and after centrifugation. Figure S6. Confocal images showing the time-dependent cellular uptake of DOX and Apt-NS-DOX by HELF cells. (A) Cellular uptake of free DOX by HELF cells. (B) Cellular uptake of Apt-NS-DOX by HELF cells. Red fluorescence indicated the DOX, green fluorescence indicated Cy5 labeled nanomaterials. Scale bar is 25 μ [file 12951_2022_1701_MOESM1_ESM.docx]

**Supporting Information**

**Self-assembly of DNA nanostructure** **containing** **cell-specific aptamer as a** **precise drug delivery system for cancer therapy in** **non-small cell lung cancer**

**Ning Wang^1#^, Chang Yu^2#^, Tingting Xu^1#^, Dan Yao^1^, Lingye Zhu^1^, Zhifa Shen^3^*, Xiaoying Huang^1^***

^1^ Division of Pulmonary Medicine, the First Affiliated Hospital of Wenzhou Medical University, Key Laboratory of Heart and Lung, Wenzhou, Zhejiang, 325000, China

^2^ Intervention Department, the First Affiliated Hospital of Wenzhou Medical University, Wenzhou, Zhejiang, 325000, China

^3^ Key Laboratory of Laboratory Medicine, Ministry of Education of China, and Zhejiang Provincial Key Laboratory of Medical Genetics, School of Laboratory Medicine and Life Sciences, Wenzhou Medical University, Wenzhou, Zhejiang, 325035, China

^#^ These authors share co-first authorship.

*** Corresponding author: Xiaoying Huang and Zhifa Shen**

E-mail address: huangxiaoying@wzhospital.cn (Huang XY); [shenzhifa@wmu.edu.cn](mailto:shenzhifa@wmu.edu.cn) (Shen ZF)

**
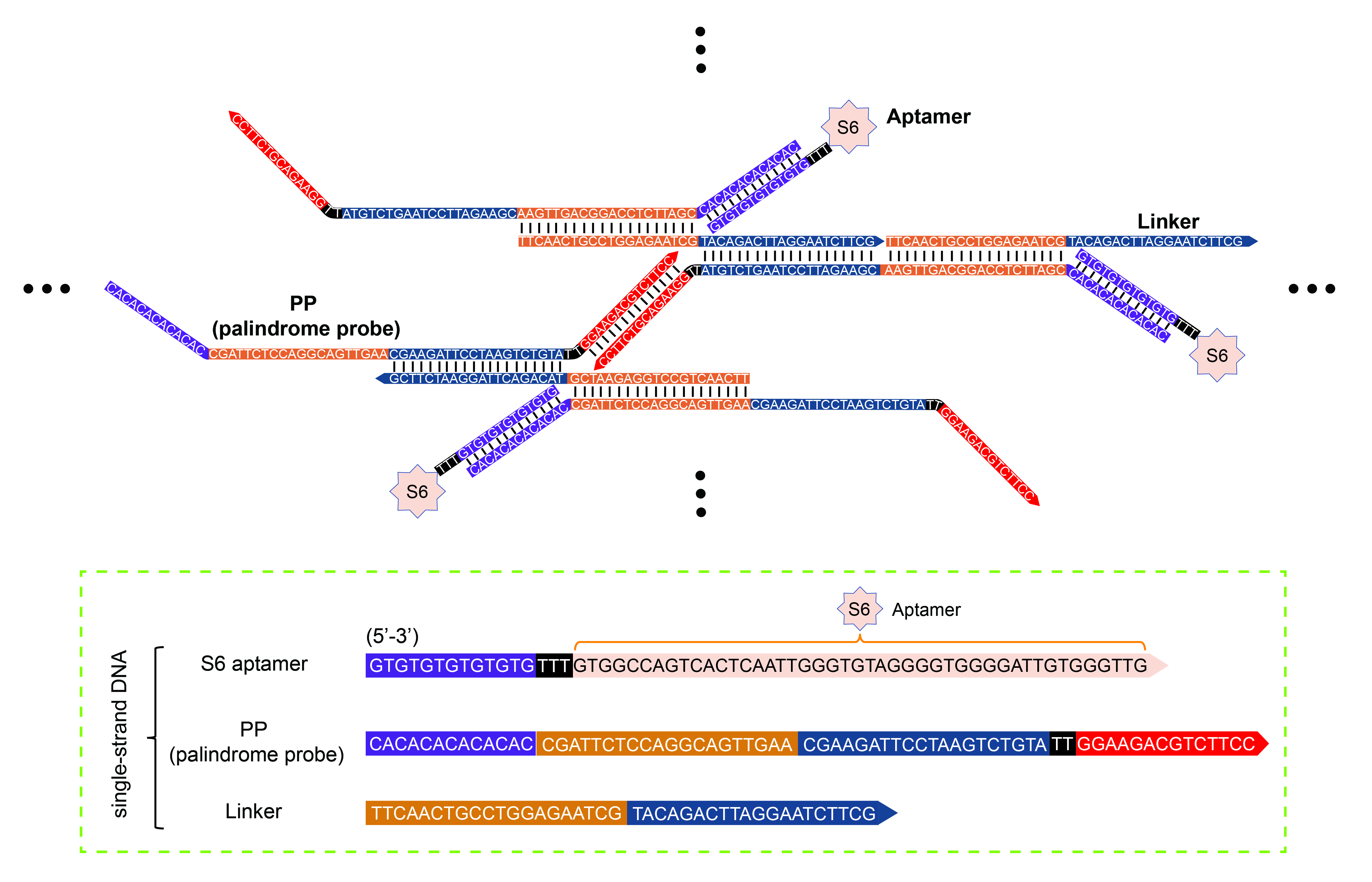
**

**Scheme S1.** The self-assembly process from single-strand DNA to a structural unit. The detail base sequences of three single-strand DNA, including S6 (aptamer), PP (palindrome probe), and Linker, are displayed in the pattern diagram.


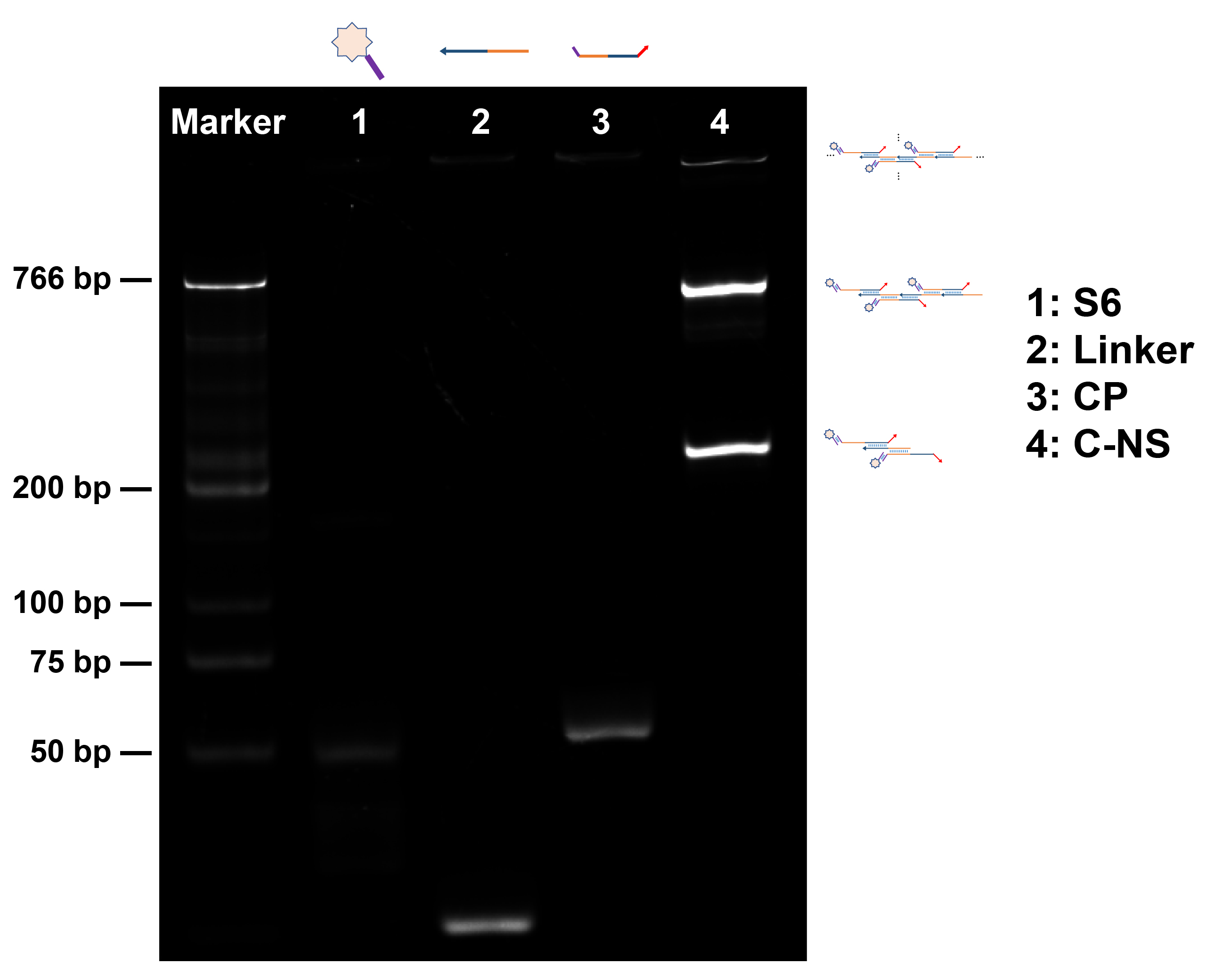


**Figure S1.** 8% PAGE image showing the influence of palindrome sequences on the synthesis of DNA nanostructure. CP (common probe) was instead of PP (palindrome probe). C-NS represented the synthetic products from self-assembly system in the absence of palindrome sequences.


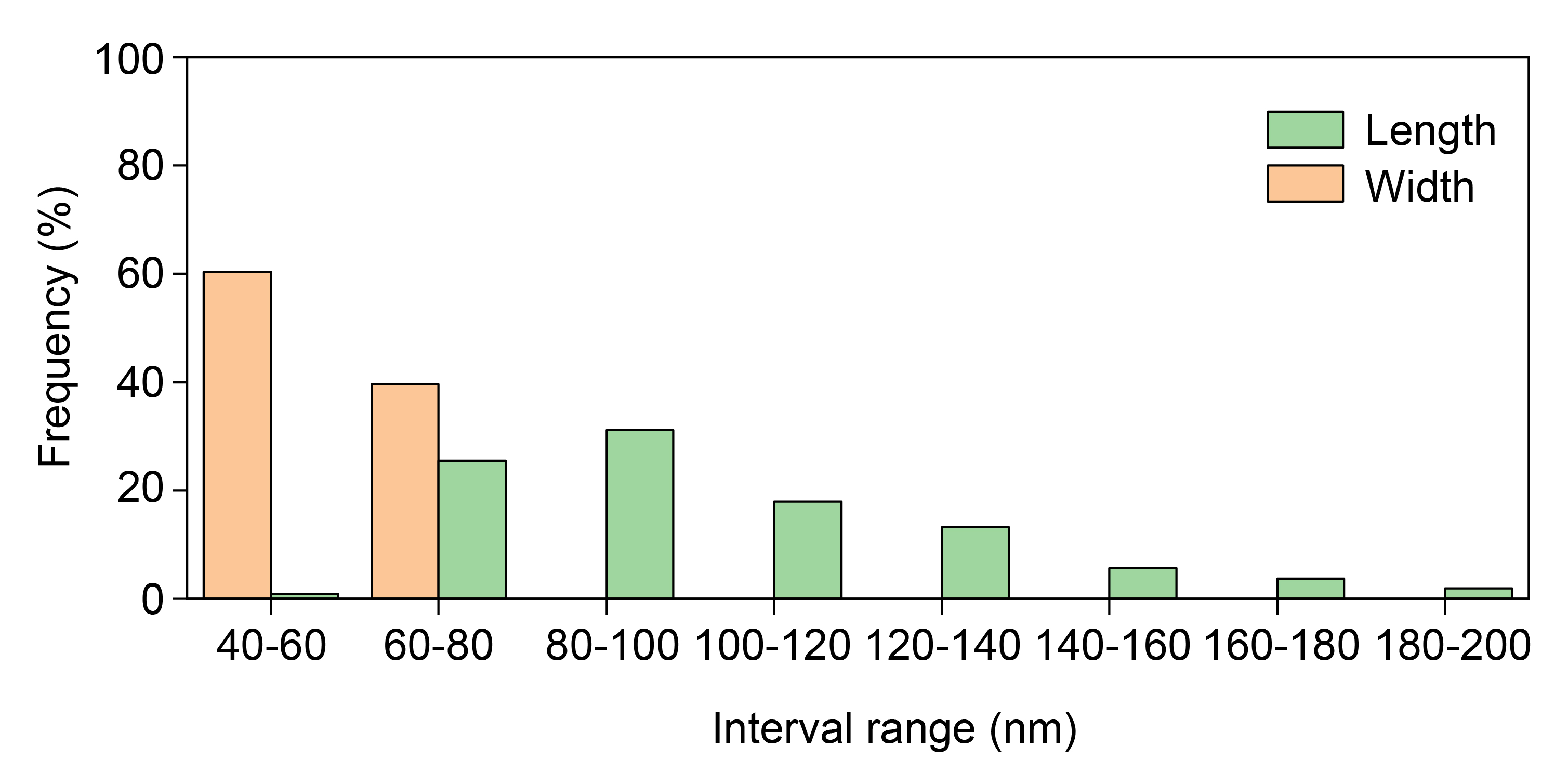


**Figure S2.** The calculation of Apt-NS sizes according to AFM images. Frequency distributions of the length and width of Apt-NS were assessed using NanoScope analysis software based on a method reported in the literature ^[1]^. The average length and width of Apt-NS were 101 ± 30 nm and 58 ± 8 nm, respectively.

[1] Zhu G, Zheng J, Song E, et al. Self-assembled, aptamer-tethered DNA nanotrains for targeted transport of molecular drugs in cancer theranostics. Proc Natl Acad Sci U S A. 2013. 110(20): 7998-8003.


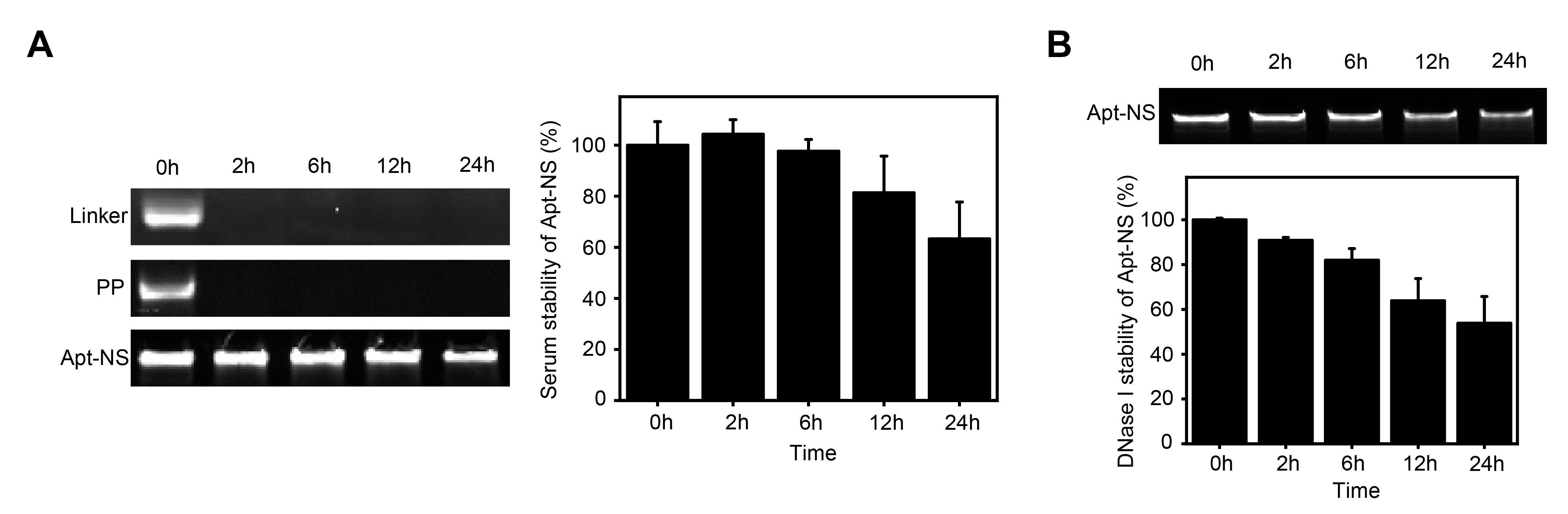


**Figure S3.** Stability assay of Apt-NS. (A) Serum stability assay of Apt-NS. PAGE analysis of samples such as Linker, PP, and Apt-NS (left panel). Quantitative assessment of Apt-NS serum stability based on gel electrophoresis images (right panel). (B) DNase I stability assay of Apt-NS. PAGE analysis of Apt-NS samples (upper panel). Quantitative assessment of Apt-NS DNase I stability based on gel electrophoresis images (lower panel).


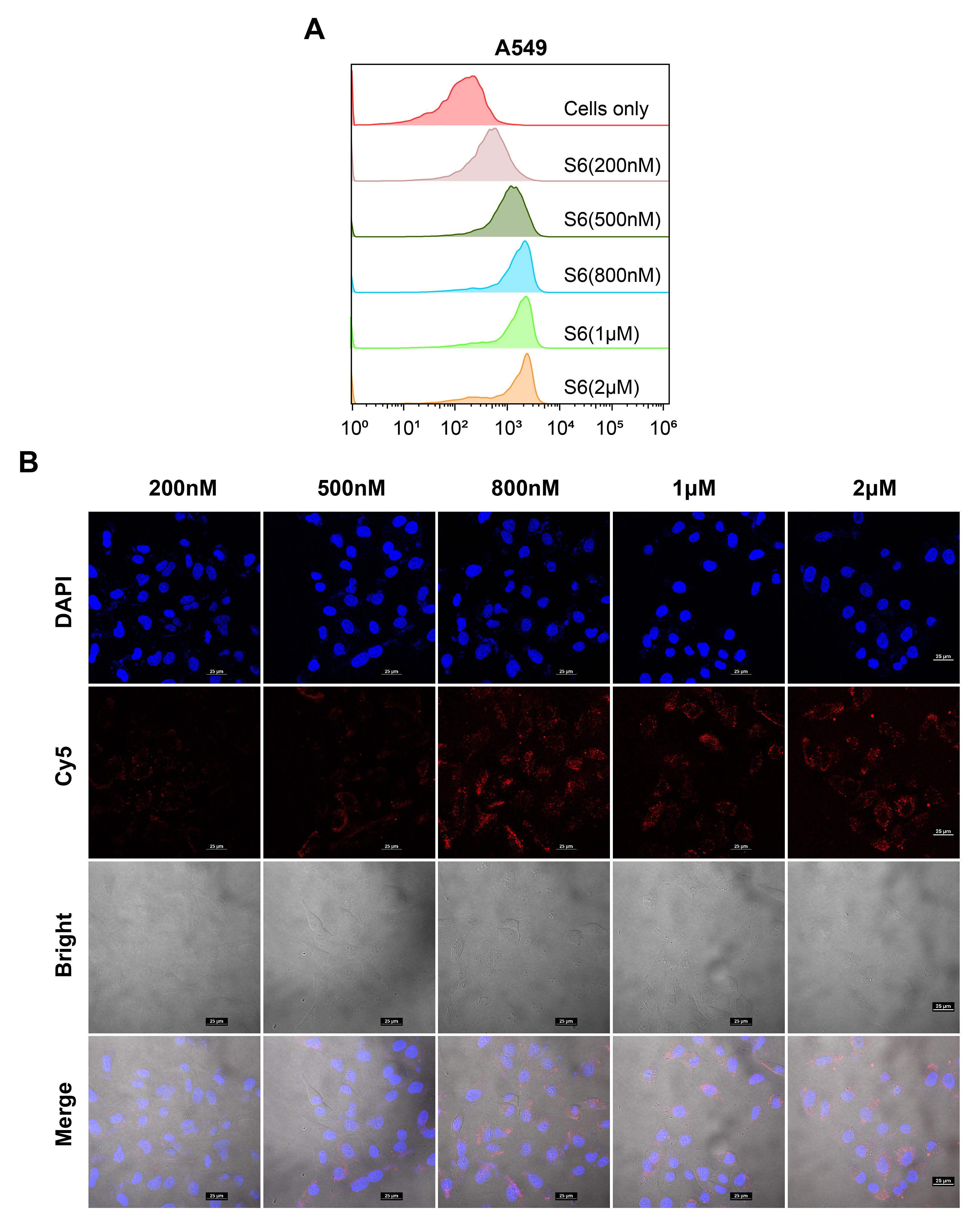


**Figure S4.** Assessment of cell recognition ability of the different concentrations of S6 aptamer on A549 cells. (A) Flow cytometric assay and (B) CLSM images showing the internalization of the different concentrations of S6 aptamer into A549 cells. Scale bar is 25 μm.


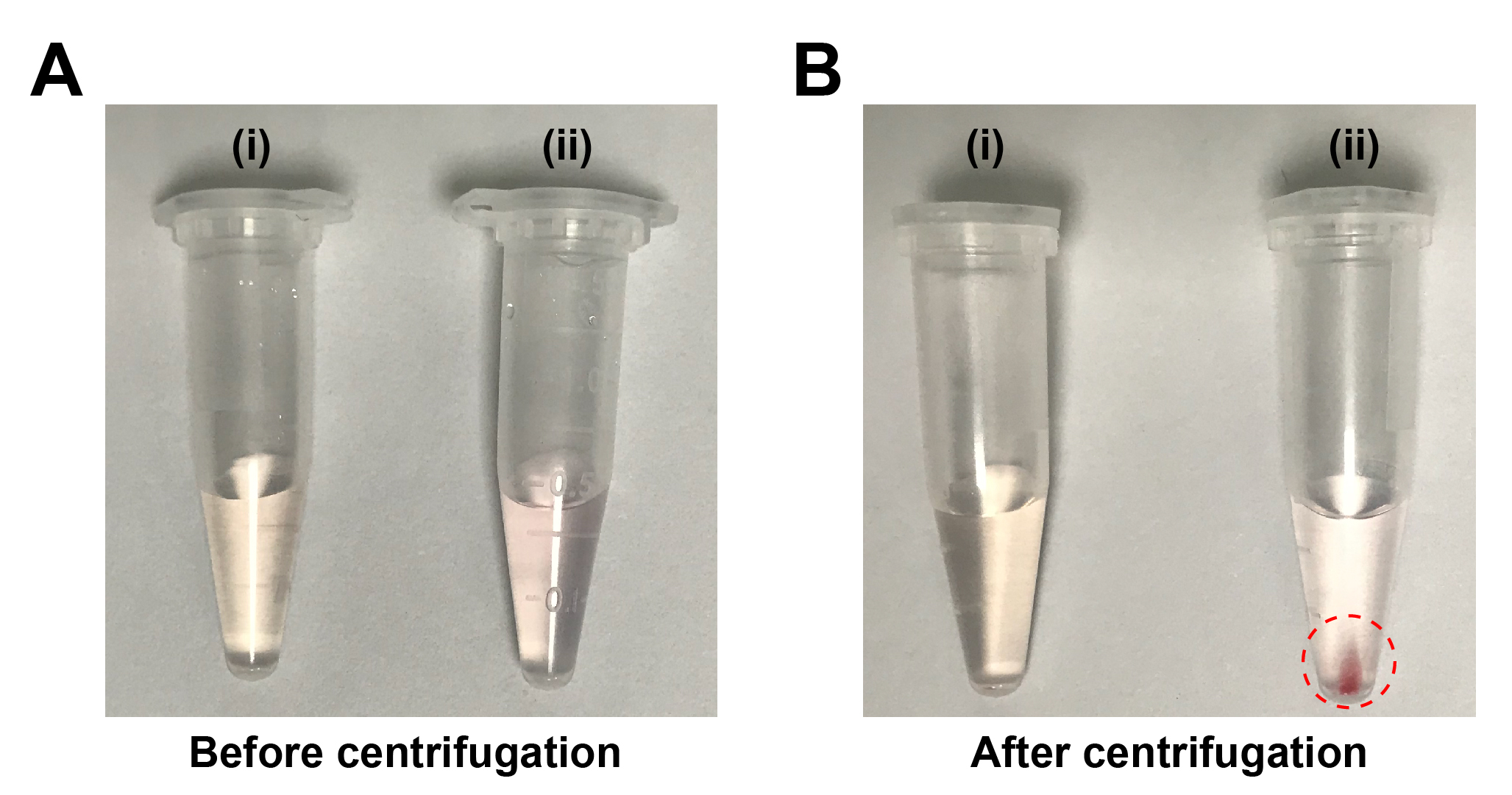


**Figure S5.** Digital photos of DOX-loaded Apt-NS. (A, B) Photographs of free DOX (i) and DOX-loaded Apt-NS solution (ii) before and after centrifugation.

Experimental procedure:

To directly observe the loading of DOX onto DNA nanomaterials, DOX (10 µM) was mixed with corresponding concentration of Apt-NS according to the molar ratio described previously at room temperature overnight. Subsequently, Apt-NS-DOX was obtained by means of centrifugation at room temperature (10 min, 10000 rpm).


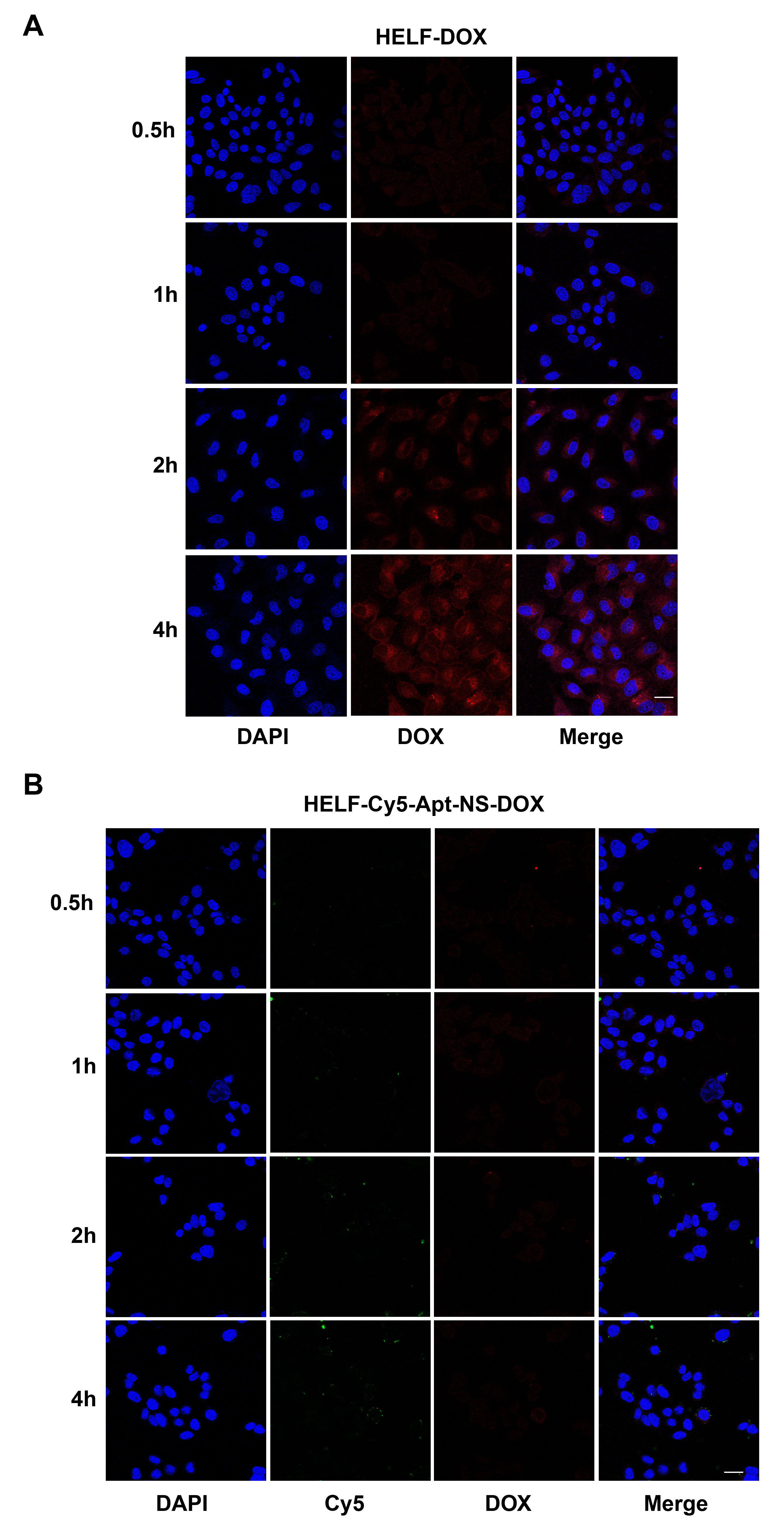


**Figure S6.** Confocal images showing the time-dependent cellular uptake of DOX and Apt-NS-DOX by HELF cells. (A) Cellular uptake of free DOX by HELF cells. (B) Cellular uptake of Apt-NS-DOX by HELF cells. Red fluorescence indicated the DOX, green fluorescence indicated Cy5 labeled nanomaterials. Scale bar is 25 μm.


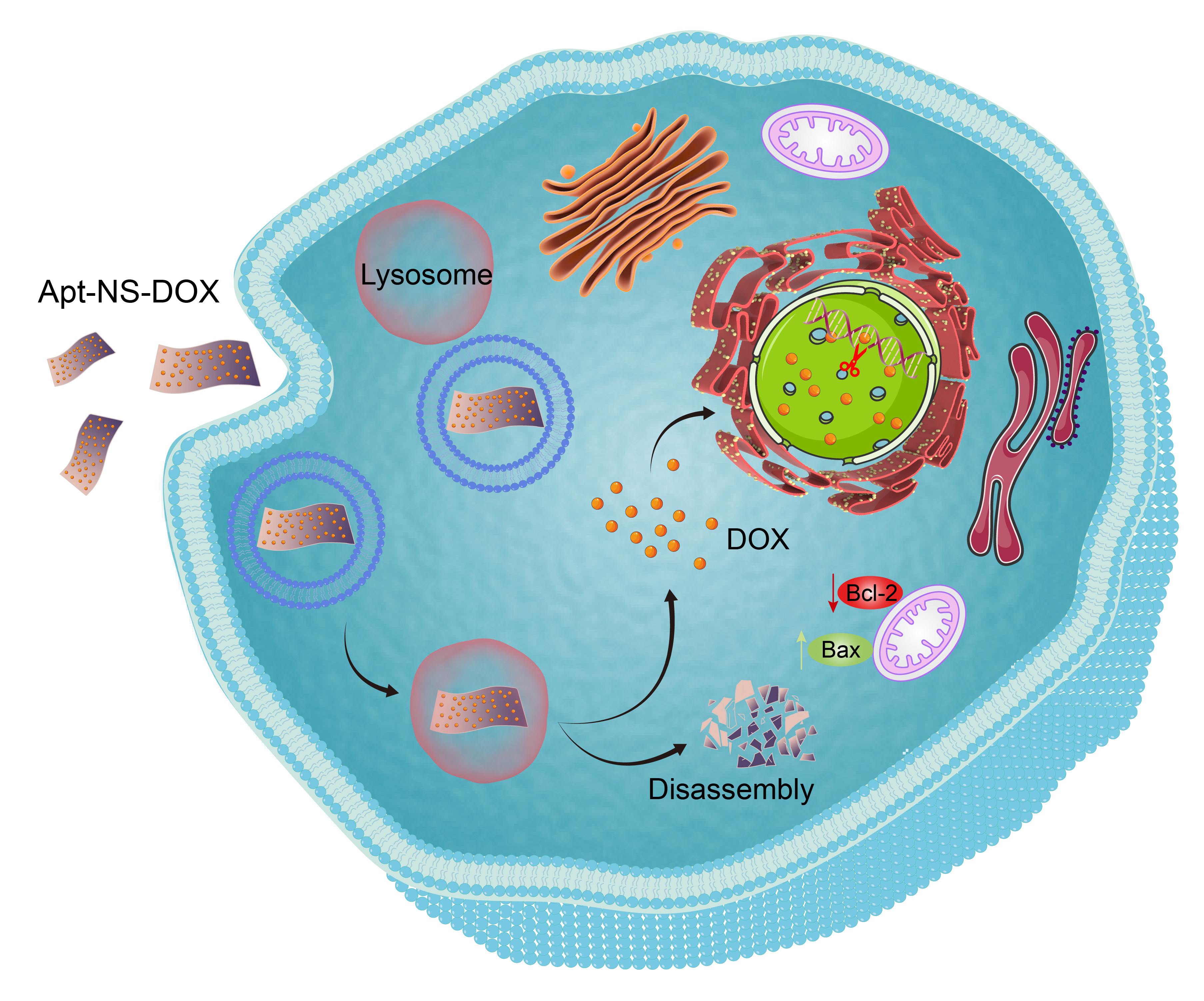


**Figure S7.** Schematic illustration of the internalization and degradation of Apt-NS-DOX in A549 cells. Internalized Apt-NS-DOX was transferred to lysosomes, and then the DOX was released by lysosomes degradation. The released DOX could enter into nucleus and induced DNA damage.


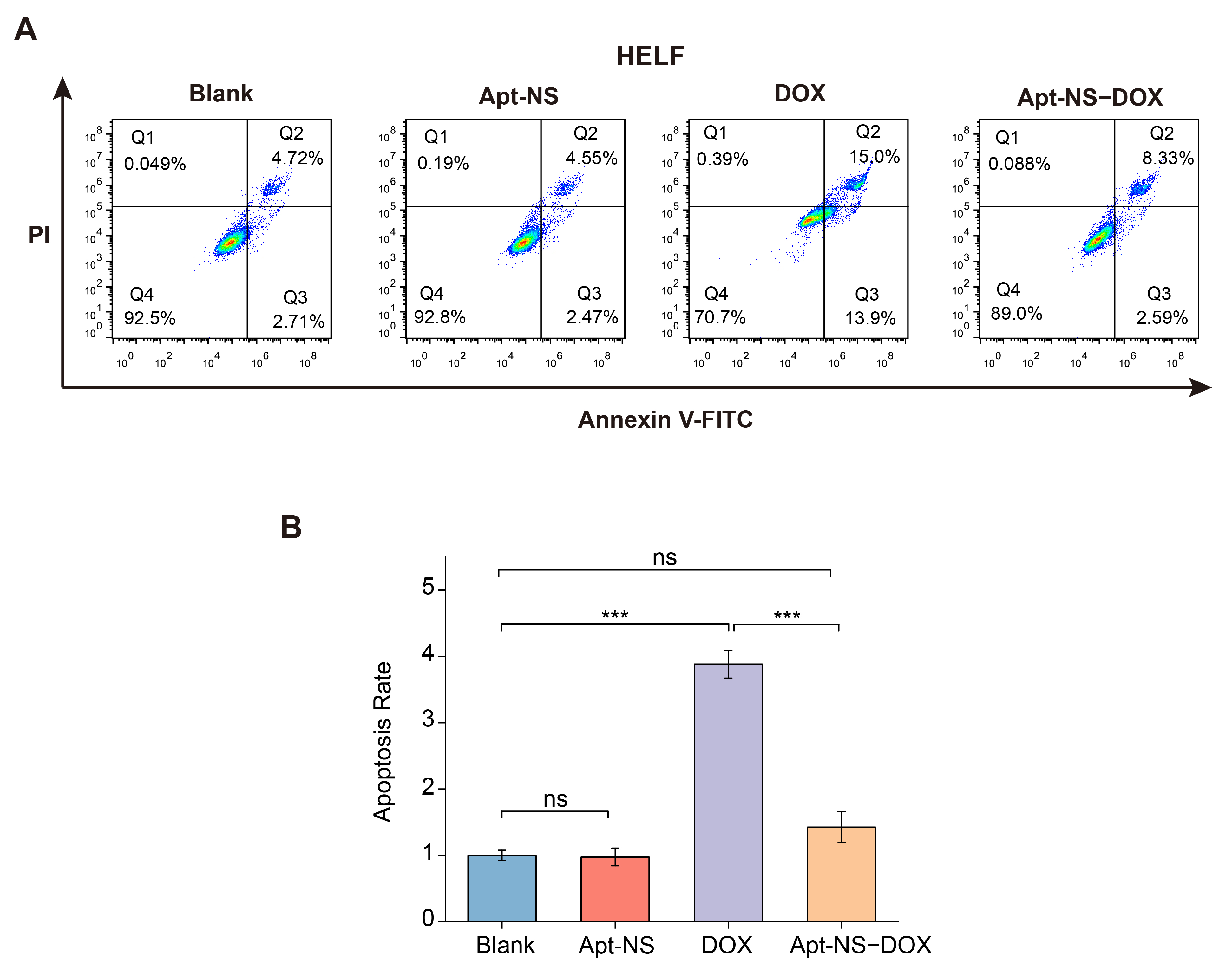


**Figure S8.** Apoptosis effects of Apt-NS-DOX on HELF cells. (A) Flow apoptosis assay of HELF cells treated with Apt-NS, DOX, and Apt-NS-DOX, respectively (Q1 means dead cells; Q2 means late apoptotic cells; Q3 means early apoptotic cells; Q4 means normal cells). (B) Quantitative analysis of cell apoptosis rate of each group in (A). Statistical analysis: ****P* < 0.001 and ns, no significance.

**
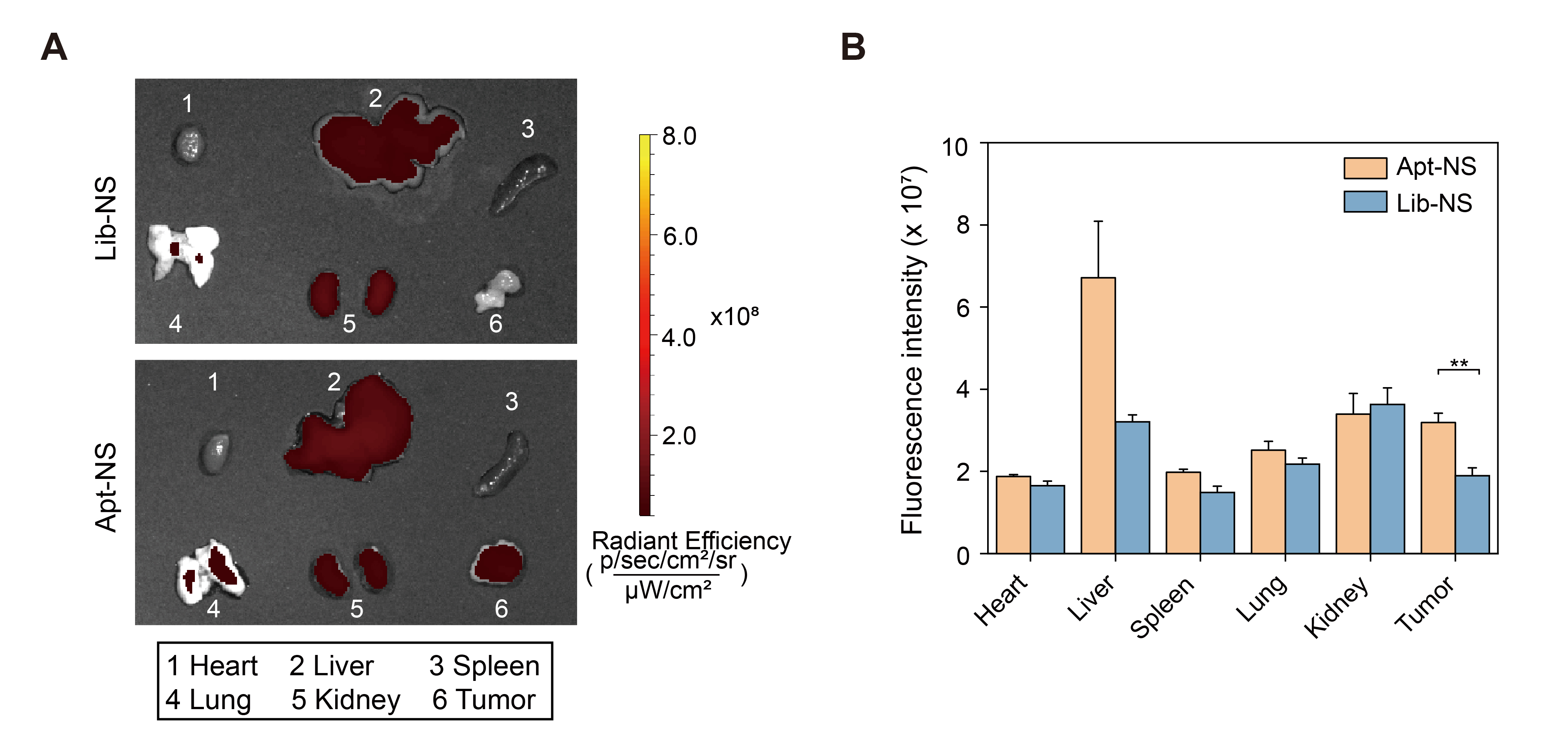
**

**Figure S9.** Nanomaterial biodistribution and tumor-targeting imaging in vivo. (A) fluorescence images of the major organs (heart, liver, spleen, lung and kidney) and tumor tissues. (B) Quantitative analysis of the average fluorescence intensities of each group in (A). Statistical analysis: ***P* < 0.01.

Experimental procedure:

**Nanomaterial biodistribution and tumor-targeting imaging in vivo**

To identify biodistribution of nanomaterials after systemic administration in the xenograft tumor model, mice were randomly divided into two groups (n = 3 in each group), and separately treated with Cy5-labeled Apt-NS and Lib-NS (Lib indicates the negative control of S6 aptamer) (Cy5-labeled ssDNA 0.1 nmol) by tail vein injections. After 6 h, the major organs (heart, liver, spleen, lung and kidney) and tumor tissues were collected to perform the fluorescence imaging for the biodistribution of the nanomaterials using IVIS-spectrum imaging system (Perkin-Elmer, USA).

**
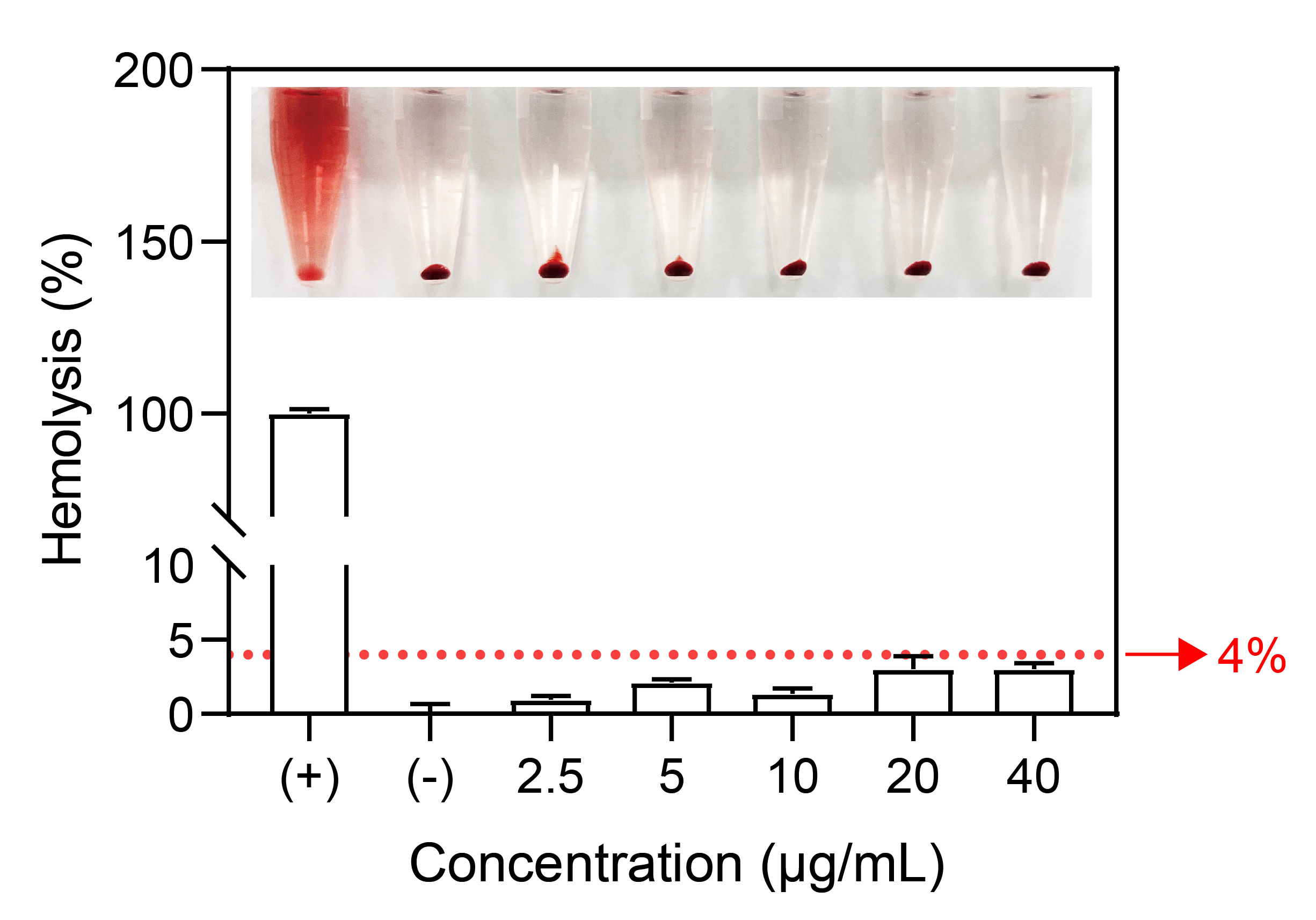
**

**Figure S10.** Hemolysis assay for drug delivery system (Apt-NS-DOX). (+) represented a positive control (H_2_O) and (-) represented a negative control (PBS). Inset: the photographs of RBCs after incubation with the corresponding samples. The value presented as mean ± SD (n = 3).

Experimental procedure:

**Hemolysis assay**

The blood samples were collected via ophthalmic vein into the anticoagulated tubes. Red blood cells (RBCs) were separated from the blood samples through the centrifugation (3000 rpm, 5 min) at 4 °C. Subsequently, the RBCs were subjected to purification with PBS. The purified RBCs were resuspend using PBS immediately. 0.2 mL of RBCs were incubated with 0.8 mL of the drug delivery systems (Apt-NS-DOX) at different concentration for 2 h at 37 °C. PBS and H_2_O were utilized as negative control and positive control, respectively. After centrifugation, the supernatant was acquired and assessed by measuring the absorbance at 541 nm ^[2]^ via a multifunctional microplate reader (Thermo, USA). The hemolysis (%) was calculated using the following formula: Hemolysis (%) = (A^sample^ - A^negative)^/(A^positive^ - A^negative^) × 100%. (A^sample^ represented the absorbance of supernatant after incubation of RBCs and nanomaterials, A^negative^ and A^positive^ represented the absorbance of supernatant in negative control (PBS) and positive control (H_2_O), respectively.)

[2] Lee H, Choi M, Kim HE, et al. Mannosylated poly (acrylic acid)-coated mesoporous silica nanoparticles for anticancer therapy. J Control Release. 2022. 349: 241-253.

**
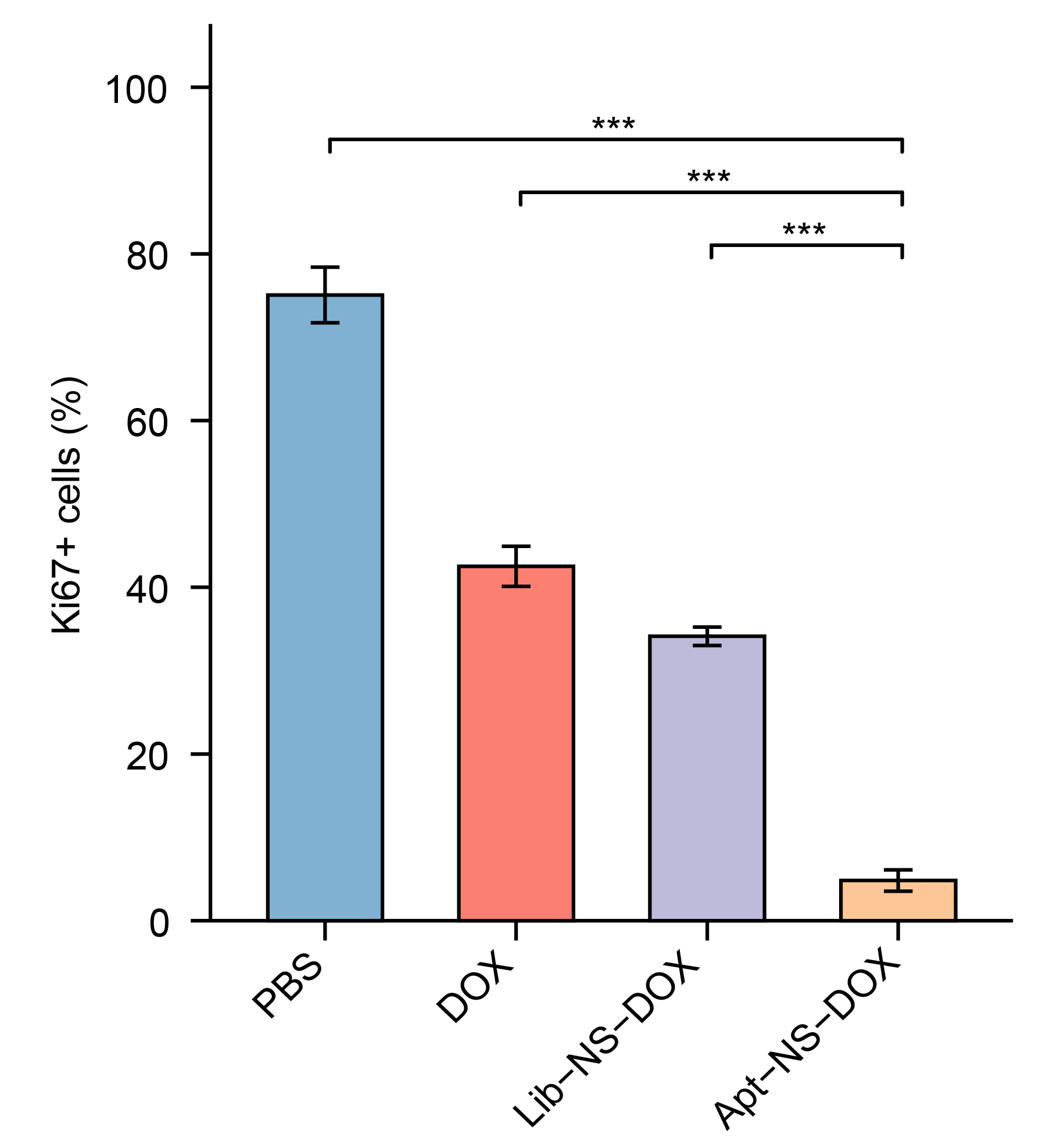
**

**Figure S11.** The calculation of Ki67-positive cells percentages according to the immunohistochemical staining of Ki67 in tumor tissues sections of different treatment groups. The value presented as mean ± SD (n = 5). Statistical analysis: ****P* < 0.001.

**
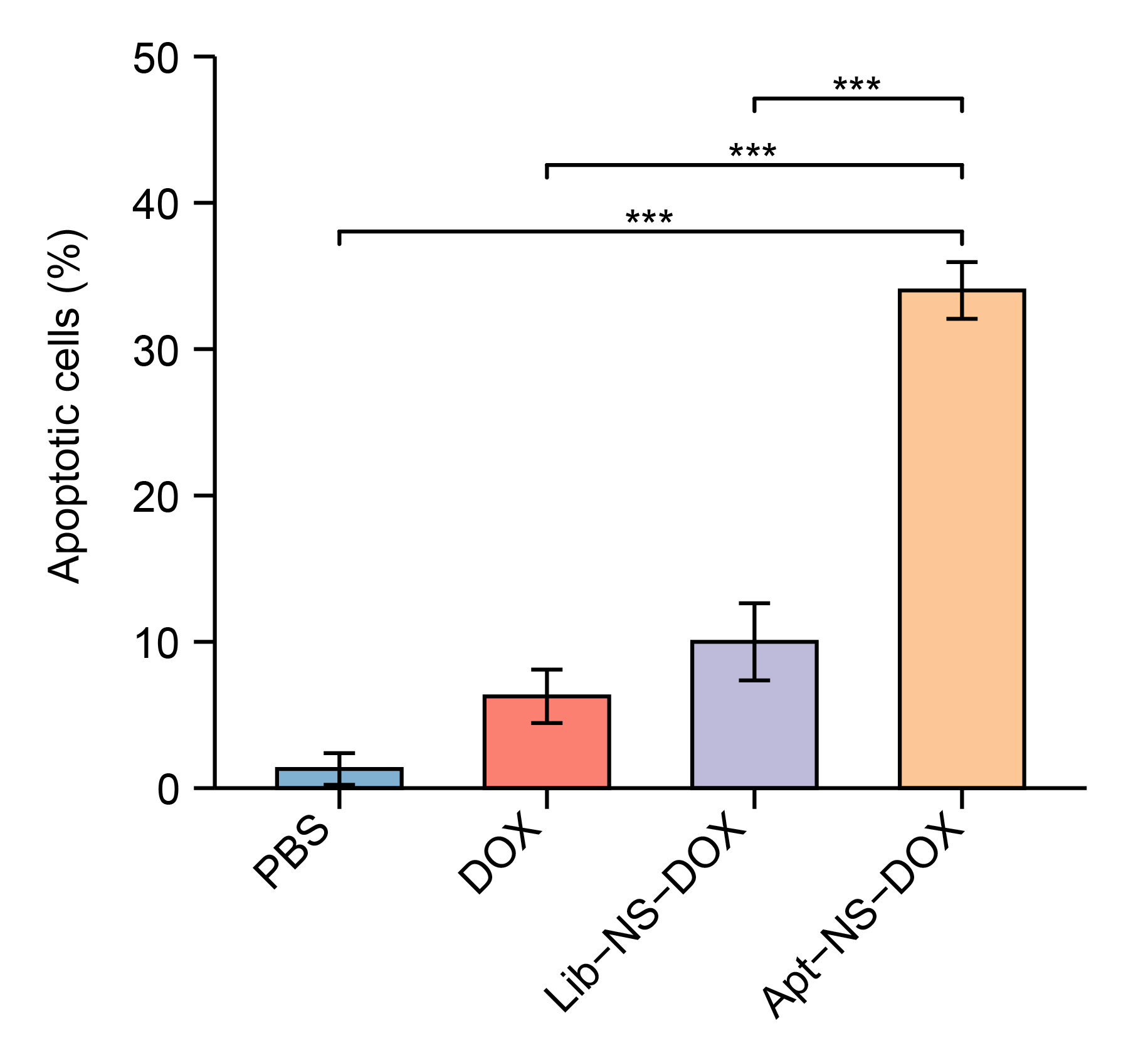
**

**Figure S12.** The calculation of apoptotic cell percentages according to the TUNEL staining of tumor tissues sections in different treatment groups. The value presented as mean ± SD (n = 3). Statistical analysis: ****P* < 0.001.

**Table S1. Sequences of ssDNA designed in our experiments**

| name | DNA sequence information (5’-3’) |
| --- | --- |
| S6 | GTGTGTGTGTGTGTTT**GTGGCCAGTCACTCAATTGGGTGTAGGGGTGGGGATTGTGGGTTG** |
| PP | CACACACACACACCGATTCTCCAGGCAGTTGAACGAAGATTCCTAAGTCTGTATT*GGAAGACGTCTTCC* |
| Linker | TTCAACTGCCTGGAGAATCGTACAGACTTAGGAATCTTCG |
| Cy5-S6 | GTGTGTGTGTGTGTTTGTGGCCAGTCACTCAATTGGGTGTAGGGGTGGGGATTGTGGGTTG-Cy5 |
| CP | CACACACACACACCGATTCTCCAGGCAGTTGAACGAAGATTCCTAAGTCTGTATT***CATAGTCCTCTTCC*** |
| Lib | GTGTGTGTGTGTGTTTNNNNNNNNNNNNNNNNNNNNNNNNNNNNNNNNNNNNNNNNNNNNN |
| Cy5-Lib | GTGTGTGTGTGTGTTTNNNNNNNNNNNNNNNNNNNNNNNNNNNNNNNNNNNNNNNNNNNNN-Cy5 |

The bold font indicates the sequence of aptamer. N indicates the negative control base of aptamer. The italic font indicates the palindromic sequence. The italic bold font indicates the negative control of the palindromic sequence. (S6: aptamer; PP: palindrome probe; Cy5-S6: Cy5-labeled S6 aptamer; CP: common probe, without palindrome; Lib: the negative control of S6 aptamer; Cy5-Lib: Cy5-labeled Lib)
